# Seasonal Variation in Transcriptomic Profiling of *Tetrastigma hemsleyanum* Fully Developed Tuberous Roots Enriches Candidate Genes in Essential Metabolic Pathways and Phytohormone Signaling

**DOI:** 10.3389/fpls.2021.659645

**Published:** 2021-07-09

**Authors:** Qianqian Xiang, Siyuan Hu, Ayalew Ligaba-Osena, Jiayao Yang, Fudan Tong, Wanli Guo

**Affiliations:** ^1^Department of Biotechnology, College of Life Sciences and Medicine, Zhejiang Sci-Tech University, Hangzhou, China; ^2^Laboratory of Plant Molecular Biology and Biotechnology, Department of Biology, The University of North Carolina at Greensboro, Greensboro, NC, United States

**Keywords:** *Tetrastigma hemsleyanum*, fully developed tuberous root, flavonoid, phenylpropanoid, starch and sucrose, transcriptome, hormone signal factor

## Abstract

*Tetrastigma hemsleyanum* Diels et Gilg (Sanyeqing, SYQ) is a perennial climbing liana and an endemic plant to southern China. Its tuberous roots (TRs) are used in traditional Chinese medicine for treating some diseases such as high fever, pneumonia, asthma, hepatitis, and cancers. However, the mechanisms underlying the development of TR and the content of flavonoids and phenylpropanoids (FPs) are not well-understood. In this study, we performed a transcriptomic analysis of 12 fully developed TR (FD-TR) samples harvested in four seasons [spring (Sp), summer (Su), autumn (Au), and winter (Wi)] using the RNA-Sequencing (RNA-Seq). We obtained a total of 78.54 Gb raw data and 65,578 unigenes. Then, the unigenes were annotated by using six databases such as non-redundant protein database (NR), Pfam, eggNOG, SWISSProt, Kyoto Encyclopedia of Genes and Genomes (KEGG), and gene ontology (GO). The transcriptomic profiling showed closer relationships between the samples obtained in Su and Au than those obtained in Sp and Wi based on the results of both total unigenes and differentially expressed genes (DEGs). Three pathways, including the biosynthesis of FPs, metabolism of starch and sucrose, and signaling of phytohormones, were highly enriched, suggesting a gene-level seasonal variation. Based on the numbers of DEGs, brassinosteroid (BR) signal transduction factors appeared to play a key role in modulating the development of TRs while most of the auxin signaling genes were mainly activated in Wi and Sp FD-TRs. Most genes in the biosynthesis and biodegradation of starch and biodegradation of cellulose were activated in Wi FD-TRs. As determined by the high performance liquid chromatography (HPLC) and aluminum nitrate colorimetric method, the contents of total flavonoids and most detected FP components increased from Sp to Au but decreased in Wi. Enhanced expression levels of some genes in the biosynthetic pathways of FPs were detected in Su and Au samples, which corroborated well with metabolite content. Our findings provide the first transcriptomic and biochemical data on a seasonal variation in the composition of medically important metabolites in SYQ FD-TRs.

## Introduction

*Tetrastigma hemsleyanum* Diels et Gilg (Sanyeqing, SYQ) is a perennial plant species belonging to the Vitaceae family. It is a diploid species (2*n* = 52) with climbing liana forming tuberous roots (TRs) during its lifecycle. SYQ is endemic to subtropical China, including Zhejiang, Fujian, Jiangxi, Yunnan, Guangxi, and Guangdong provinces (Wang Y. H. et al., 2015; Guo et al., 2019), and is not resistant to low temperature (Peng et al., 2019). Its TRs are mainly used as Chinese folk medicine, which was recorded first in the book *Compendium of Materia Medica* (Li Shizhen, 1815–1893, Chinese). SYQ TRs are rich in phenylpropanoids, flavonoids, and polysaccharides (Guo et al., 2019; Zhu et al., 2020). Therefore, SYQ TR has some pharmacological activities such as the elimination of phlegm, improving blood circulation, detoxification, antipyretic (Yang and Wang, 2013), analgesic, anti-inflammatory (Huang et al., 2005), and antiviral (Yang et al., 1989; Yang and Wu, 2009), and is applied for treating children with high fever, infantile febrile convulsion, pneumonia, asthma, hepatitis, rheumatism, irregular menstruation, sore throat, scrofula, snakebite, and jaundice (Guo et al., 2019). Recently, SYQ TR is known to have an antitumor activity with no or little cytotoxicity to normal cells (Chen et al., 2017) in leukemia and carcinomas of the lung, liver, stomach, and intestine (Guo et al., 2019; Zhu et al., 2020) by improving the immune system (Xu et al., 2008) and antioxidants (Ye and Liu, 2015). However, little is known about the mechanisms of TR growth, development, and metabolisms of the valuable components in SYQ.

The development of TRs and tubers is a complex process, which depends on the balances between endogenous factors, such as phytohormones, sucrose, and starch, and exogenous environmental factors, including temperature, drought, photoperiod, and nutrient status (Khan et al., 2016; Utsumi et al., 2020). The mechanism of tuberous organ formation has been widely studied in root crops such as sweetpotato (*Ipomoea batatas*), potato (*Solanum tuberosum*), and cassava (*Manihot esculenta*) (Tanaka, 2016; Hoang et al., 2020), indicating the role of endogenous phytohormones, sucrose, and starch in the development of TR. Cytokinins (CTKs) (Tanaka et al., 2008; Sojikul et al., 2015), auxins (Faivre-Rampant et al., 2004; Noh et al., 2010; Roumeliotis et al., 2012; Sojikul et al., 2015), jasmonic acid (JA) (Koda et al., 2001; Cenzana et al., 2003), and brassinosteroids (BRs) (Wei and Li, 2016; Que et al., 2018; Hoang et al., 2020) improve the initiation of TR. However, the level of auxin has been shown to decrease gradually during the secondary thickening growth of the TR (Tanaka et al., 2008; Noh et al., 2010; Dong et al., 2019; Ding et al., 2020). The expansion of TRs is positively correlated with abscisic acid (ABA) and CTKs (Wang Q. et al., 2006; Sun et al., 2015). Ethylene also stimulates root tip swelling by inhibiting root elongation through blocking gibberellins (GA) biosynthesis (Van De Poel et al., 2015). Indeed, GA, an antagonist of ABA and ethylene, has been shown to inhibit the formation of TR but promotes stolon, root, and stem elongation (Kloosterman et al., 2007). These phytohormones also interact with each other through their signal transduction factors. For example, CTKs and JA induce the expression of a MADS-box gene *IbMADS1* during the early stage of initiation of TR in sweetpotato (Ku et al., 2008), and indole-3-acetic acid (IAA) regulates another MADS-box gene *SRD1* to activate the proliferation of cambium and metaxylem cells and consequently improve initial thickening of TRs (Noh et al., 2010). The phosphorylation of an ABRE binding factor ABA-responsive element binding factor 1 (ABF1) protein is inhibited by GA (Muñiz García et al., 2012) in potato, but ABF4 upregulates *StGA2ox1* (GA inactivated gene) to improve tuberization in the stolon subapical region (Kloosterman et al., 2007; Muñiz García et al., 2014, 2018). However, little is known about the roles of these phytohormones and their signal transduction factors in the modulation of TRs in SYQ.

The accumulation and metabolism of starch and sucrose form the basis of plant growth and development and play an important role in the development of TRs (Wang S. et al., 2006; Villordon et al., 2014; Hoang et al., 2020). Starch is one of the main storage compounds in TR, and some genes in starch metabolism, including those encoding β-amylase, ADP-glucose pyrophosphorylase (AGPase), α-1–4 glucan phosphorylase, and starch synthase (SS) (Wang S. et al., 2006; Firon et al., 2013; Ponniah et al., 2017), have been shown to be upregulated during the initiation and growth of TR (Firon et al., 2013; Villordon et al., 2014). For example, an increase in the level of *AGPase* has been shown to enhance biomass and yield in cassava tubers (Ihemere et al., 2006). Sucrose is not only one of the main photoassimilates but also a driving force for the formation and growth of TR (Ovono et al., 2009). It has been reported that a high sucrose level is required for *in vitro* induction of TR in sweetpotato (Tsubone et al., 2000). Accordingly, genes in sucrose synthesis (SUS) (Wang et al., 1993) and sucrose-phosphate synthase (SPS) (Tao et al., 2012) were upregulated in radish (*Raphanus sativus*) TR (Mitsui et al., 2015). Sucrose also plays an important role in signaling by interacting with other signal transduction factors in modulating the architecture of TR and biosynthesis and perception of hormones such as ABA (Rolland et al., 2002; Koch, 2004) and CTKs (Eguchi and Yoshida, 2008; Tanaka et al., 2008). In addition, CTKs also regulate the biosynthesis of starch and differentiation of plastids from proplastids to amyloplasts (Enami et al., 2011). These reports suggest that CTKs, JA, ABA, BRs, ethylene, GA, sucrose, their signal transduction factors, and the accumulation and metabolism of starch are involved in the formation of TR.

The shape of SYQ TRs is calabash, spindle, ovate, etc., and they are linked as a necklace (Wang et al., 2015a). Like sweetpotato (Ravi et al., 2014), SYQ TR emerges from the swelling of adventitious roots, which are developed from cut ends, nodal primordia, or wounds of stems in 5–15 days after transplanting (unpublished data). However, TRs in sweetpotato are larger in size than those of SYQ, and only grow for several months before harvesting while SYQ TRs are smaller and grow for more than 3 years before harvesting and being used as medicine (Guo et al., 2019). Thus, it is difficult to study the mechanisms of growth and development and metabolite changes of SYQ TRs.

RNA-Sequencing (RNA-Seq) has become a powerful and cost-effective tool for exploring the molecular mechanisms regulating TR development and metabolic pathways of medicinal components (Sun et al., 2015; Chaudhary and Sharma, 2016; Dong et al., 2019). RNA-Seq allows the discovery of novel genes in various biological processes, quantification of gene expression, and identification of genetic variation, and is also suitable for many non-model organisms (Chaudhary and Sharma, 2016). Thus, the RNA-Seq has been developed rapidly over the last decade to illustrate biological processes such as the regulatory mechanism of storage root development in sweetpotato (Firon et al., 2013; Dong et al., 2019; Li et al., 2019), cassava (Ding et al., 2020; Utsumi et al., 2020), *Rehmannia glutinosa* (Li et al., 2015), and *Callerya speciosa* (Xu et al., 2016). RNA-Seq is also a powerful tool to study the biosynthesis pathways of secondary metabolites in TRs and tubers (Wang et al., 2017; Wu et al., 2017) such as flavonoids and phenylpropanoids (FPs) in *Pueraria lobata* (Wang et al., 2015b); saponin in *Panax* (Rai et al., 2016; Lee et al., 2017); and alkaloids in *Corydalis yanhusuo* (Liao et al., 2016). Wang Y. H. et al. (2015) and Wang et al. (2018) used RNA-Seq and chloroplast DNA sequencing to integrate multiple nuclear loci for phylogeographic and phylogenetic diversities of SYQ populations. Peng et al. (2019, 2020) studied the transcriptome, endogenous hormones, and unigenes in flavonoid biosynthesis in SYQ leaves in response to short-time cold while Yan et al. (2020) summarized the integration of transcriptome and metabolome of SYQ purple and green leaves and discussed the expression patterns of some genes in the anthocyanin biosynthesis pathway. However, those prior studies mainly focused on SYQ leaves not on the main medicinal organ TRs.

**Figure 1:**
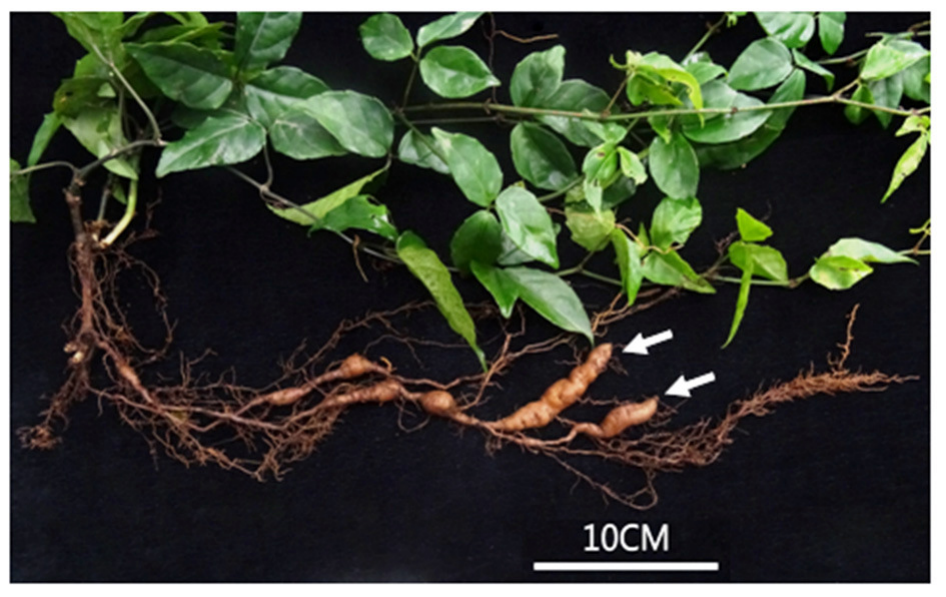
Phenotype of a 28-month-old *T. hemsleyanum* plant. Arrows indicate fully developed tuberous roots (FD TRs).

In this study, we analyzed the transcriptomic changes in response to a seasonal variation in a fully developed TR (FD-TR, Figure 1) of *T. hemsleyanum*. (1) Transcriptomic library was constructed from the 12 FD-TR samples, and the differentially expressed genes (DEGs) were analyzed according to the sampling seasons. (2) DEGs were enriched for the three main pathways of biosynthesis of FPs, phytohormone signaling, and sucrose and starch metabolism. (3) The content of total flavonoids was measured by using the standard procedures, some components of FPs were detected by the high performance liquid chromatography (HPLC), and the correlations between the contents of components and the expression levels of its biosynthetic genes were observed. To our knowledge, this is the first global transcriptomic analysis to identify the genetic elements regulating the development of TR and a seasonal variation of some contents of FPs in SYQ. These findings may improve our understanding of the molecular basis of TR development and metabolite accumulation in *T. hemsleyanum*.

## Materials and Methods

### Plant Materials and Growth Conditions

Commercial SYQ TRs are usually harvested 3 years after transplanting stem cutting. In this study, plants were cultivated under natural growing conditions to mimic traditional cultivation at Hangzhou Sanyeqing Agricultural Technology Co., LTD., Hangzhou, China (N: 35°3′31″, E: 113°7′17″, elevation: 500–700 m). FD-TRs were obtained in 2016 from the SYQ plants grown for 28 months (Figure 1). FD-TRs were sampled in four seasons such as spring (Sp, April 30, when the soil surface temperature (*T*_*m*_) was 24°C), summer (Su, July 24, 37°C, the shoot growth of SYQ plants is faster in Su than other seasons), autumn (Au, September 26, 29°C, SYQ plants accumulate storage metabolites, including starch, protein, and secondary metabolites in TR), and winter (Wi, December 5, 12°C). FD-TRs have a relatively stable phenotype and grow slowly after the first 2 years (Figure 1). To guarantee uniformity among the samples, sampling times were scheduled from 9:00 to 12:00 a.m. in more than three sunshine days after rain or irrigation. Five replicates were obtained at each sampling time, and five randomly selected plants were used for each replicate. FD-TRs in each replicate were immediately washed thoroughly; cut into small pieces, well mixed, and put in 50-ml plastic tubes quickly; frozen in liquid nitrogen; and then stored at −80°C until use.

### RNA Isolation and Sequencing

Total RNA was isolated from the 12 samples by using the Trizol reagent (Invitrogen, CA, USA) following the manufacturer's procedure. Three out of five replicates at each sampling time were selected based on similarity in the contents of total flavonoids. The total RNA quantity and purity were determined by using the RNA 6000 Nano Lab Chip Kit and Bioanalyzer 2100 (Agilent, CA, USA) with RIN number > 7.0. Poly (A) messenger RNA (mRNA) was isolated from a 10-μg total RNA by using poly-T oligo attached magnetic beads (Invitrogen, CA, USA). Following purification, the poly (A)-RNA fractions were fragmented into small pieces by using divalent cations under elevated temperatures. Then, the cleaved RNA fragments were reverse transcribed to create the final complementary DNA (cDNA) library according to the mRNA-Seq sample preparation kit (Illumina, San Diego, CA, USA), the average insert size for the paired-end libraries was 300 bp (±50 bp). Then, we performed paired-end sequencing on an Illumina Hiseq 4000 (LC-Bio, Hangzhou, China) following the vendor's recommended protocol.

### *De novo* Assembly, Annotation, and Functional Classification

The raw reads were filtered for an adapter, ambiguous residues, and low-quality reads prior to a transcriptomic assembly by using a perl script Cutadapt v2.10 (Martin, 2011). Then, the sequence quality was verified by using FastQC v0.11.9 (http://www.bioinformatics.babraham.ac.uk/projects/fastqc/). Filtered reads of 12 libraries were pooled for *de novo* assembly of the global transcriptome by using Trinity v2.4.0 (Grabherr et al., 2011). The coding regions within unigenes were predicted by TransDecoder v5.5.0 (https://github.com/TransDecoder). All assembled unigenes were aligned against the databases, including non-redundant protein database (NR) (http://www.ncbi.nlm.nih.gov/), gene ontology (GO) (http://www.geneontology.org), SwissProt (http://www.expasy.ch/sprot/), Kyoto Encyclopedia of Genes and Genomes (KEGG) (http://www.genome.jp/kegg/), Pfam (http://pfam.xfam.org/), and eggNOG (http://eggnogdb.embl.de/), for gene annotation by using the DIAMOND software v0.8.3.8 (Buchfink et al., 2015), and unigenes with the threshold value of *e* ≤ 10^−5^ and *p* < 0.05 were considered to be significantly enriched in GO and KEGG analyses (a hypergeometric test was used). Venn diagrams were generated by using the online software Venny 2.1 (http://bioinfogp.cnb.csic.es/tools/venny/). The RNA-Seq reads were deposited in the NCBI Sequence Read Archive (SRA, http://www.ncbi.nlm.nih.gov/Traces/sra) under BioProject accession number PRJNA703059.

### Analysis of Differentially Expressed Genes

After the assembly and annotation, clean reads of the 12 libraries were mapped to the global transcriptome by using the software Salmon v1.0.0 (Patro et al., 2017), and the number of mapped clean reads per unigene was calculated and normalized to the number of reads per kilobase per million (RPKM) (Mortazavi et al., 2008). DEGs were selected with |log_2_ (fold change, FC) ≥ 1 and with statistical significance (value of *p* < 0.05) by using edgeR v1.0.0 in R package (Robinson et al., 2010). Meanwhile, a gene expression level (RPKM) with the false discovery rate (FDR < 0.05) was scaled by using the *Z*-score with the formula: *Z* = (*X*–μ)/σ before a clustering analysis, where *X* represents the RPKM of a gene in a specific sample/time point, and μ and σ are the mean transcript expression and SD of a gene across all samples, respectively (Mou et al., 2015). The normalized values were used to analyze the gene expression patterns (heatmap) among different samples by using the software TMeV v4.9.0 (Saeed et al., 2003).

### Extraction and Quantification of Total Flavonoids

To determine the content of total flavonoids in the FD-TR samples, five biological replicates prepared in the previous section “*Plant materials and growth conditions*” at each sampling time were used, and from each biological replicate, three technical replicates were prepared. Each technical replicate was transferred to a 50-ml plastic tube and stored at −80°C, and was freeze dried at −20°C by using Eppendof Concentrator for 2 days. Freeze-dried roots were ground by using a mortar and pestle to obtain a fine powder (100-mesh sieve). From this powder, 0.3 g representative sample weight from each technical replicate was extracted with 3 ml of 65% ethanol (v/v) at 25°C for 60 min by using an ultrasonic wave (200 W, 20 kHz), and the extraction solution was centrifuged at 2,057 × g for 10 min to collect the supernatant (Eppendorf Centrifuge 5804R, Hamburg, Germany) using an extraction process, which was repeated three times. The insoluble residue was washed with three rounds of 65% ethanol, and the final volume was adjusted to 25 ml. The solution was filtrated through a 0.2-μm filtration membrane. Total flavonoids were estimated by using the aluminum nitrate [NaNO_2_-Al(NO_3_)_3_-NaOH] colorimetric method with rutin (a common dietary flavonoid) as a standard for estimating the content of total flavonoids with some modifications (Ye and Liu, 2015). Briefly, 2 ml filtrated solution, 4 ml of 65% ethanol, and 1 ml of 5% sodium nitrite (w/v) were gently mixed and incubated for 6 min at 25°C. Then, 1 ml of 10% (w/w) aluminum nitrate was added and mixed, and 10 ml of 5 N NaOH was added and mixed after 6 min. The total volume was adjusted to 25 ml by adding 65% ethanol. Finally, the solution was incubated for 15 min and centrifuged at 2,057 × g for 10 min, and the absorbance OD_500_ of the supernatant was measured. A calibration curve was generated by varying the concentration of rutin, including 0, 200, 400, 600, 800, and 1,000 μg/ml in a volume of 1 ml double-distilled water. Multiple linear regression method in SPSS 19.0 software (IBM, Armonk, NY, USA) was used to *g*enerate the linear regression equation determining the content of total flavonoids (*y* = 10.871 *x*−0.0045, *y* is the absorbance at OD_500_, *x* is the content of total flavonoids, linear range is 8–48 μg/ml, and *R*^2^ = 0.9972).

### Quantification of Some FP Components

To quantify the composition of FPs such as protocatechuic acid, catechin, chlorogenic acid, and others, 0.3 g of TR powder prepared in the previous section “*Extraction and quantification of total flavonoids*” was extracted once with 6 ml of 70% (v/v) methanol for 3 h under an ultrasonic wave (200 W, 20 kHz) at 25°C. The extract was then centrifuged at 2,057 × g for 15 min, and the supernatant was filtered by using 0.22-μm membrane filters. The filtrate was analyzed by using the HPLC to quantify the level of 14 compounds, including protocatechuic acid, catechin, chlorogenic acid, orientin, polydatin, isoquercitrin, nictoflorin, piceatannol, astragaline, resveratrol, kaempferol, myricetin, quercetin, and naringenin. Standard of each compound was prepared from the pure compounds purchased from the National Institute for Control of Pharmaceuticals and Biological Products (Beijing, China). Calibration curves were generated for each compound from the solutions containing varying concentrations of pure compounds (0.1, 0.5, 2.5, 10, 20, 40, and 60 μg/ml) by using a multi-linear regression tool available in SPSS 19.0.

An analysis of target compounds was carried out by using Waters e2697 and a photodiode array detector 2998 (Waters Corp., Milford, MA, USA). The HPLC was performed with Waters SunFire® C18 column (250 × 4.6 mm, 5 μm) at 30°C. About 0.1% methanoic acid (A, w/v) and acetonitrile (B) were used as the mobile phase at a flow rate of 0.8 ml/min: 95–85% A (v/v) within 0–20 min; 85–66% A within 20–58 min; 66–63% A within 58–65 min; 63–30% A within 65–75 min; and 30–0% A within 75–76 min. The injection volume was 20 μl, and the compounds were detected at wavelengths of 250, 270, 280, 315, and 370 nm.

### Quantitative PCR

The expression of candidate unigenes was validated by using quantitative PCR (qPCR). Total RNA was isolated from the 12 TR samples as described above in the section “*RNA isolation and RNA-Sequencing*”. First-strand cDNA was synthesized by using Transcript One-Step gDNA Removal (gDNA wiper Mix, Vazyme Biotech Co., Nanjing, China) and cDNA HiScript II qRT SuperMix II (Vazyme) following the manufacturer's instructions. Gene-specific primers for quantitative real-time- (qRT-) PCR were designed by using the real-time PCR (TaqMan) Primer and Probes Design tool (https://www.genscript.com/tools/) and listed in Supplementary Table 1. PCR reactions were performed by using the ABI 7,500 real-time PCR detection system (Applied Biosystems) with the ChamQ Universal SYBR qPCR Master Mix (Vazyme). The volume of each reaction was 10 μl, which contains 2 μl cDNA, 5 μl 2x ChamQ Universal SYBR qPCR Master Mix, and forward and reverse primers (each 0.2 μl from a 10-μm stock). The qPCR program was 95°C for 30 s, 30 cycles of 95°C for 30 s, and 60°C for 60 s, followed by a melting curve analysis. Relative gene expression levels were calculated by using the 2^−ΔΔCt^ method (Livak and Schmittgen, 2001) with three biological replicates. The expression of four housekeeping genes was compared to select an optimal internal reference (Supplementary Table 1).

### Statistical Analysis

Spearman's non-parametric correlations and Pearson correlation (Hollander and Wolfe, 1973) were performed by using the SPSS Software 19.0. Significant differences were determined by ANOVA according to the Studentized range Q method (Snedecor and Cochran, 1967).

## Results and Discussion

### Characteristics of RNA-Seq and *de novo* Assembly

To study the transcriptomic changes between FD-TRs of *T*. *hemsleyanum* harvested in the four seasons (Sp, Su, Au, and Wi), cDNA libraries were generated from the three biological replicates and sequenced by using the Illumina Hiseq 4000. A total of 520,071,202 (78.54 Gb) raw reads with an average of 43,339,267 (6.55 Gb) raw reads were obtained for each sample. After filtering, 507,874,152 (75.65 Gb) clean reads with 42,322,846 (6.30 Gb) reads per sample were obtained. High percentages of clean reads/raw reads with a high average percentage of Q20 (the sequencing error rate is < 0.01) and Q30 (the sequencing error rate is < 0.001) and reasonable CG contents (average 47.35 and 47.43% for clean reads and raw reads, respectively), were obtained, which validate the sequence quality (Supplementary Table 2). The total clean reads of the 12 libraries were subsequently *de novo* assembled, resulting in 144,096 transcripts with an N50 value of size 1,627 nt and median size 552 nt; and 65,578 unigenes with an N50 value of 1,450, and the median size was 393 nt (Table 1). The distributions and GC contents of 65,578 unigenes and 144,096 transcripts were both similar to a Gaussian distribution model, and the highest frequencies were 41% GC for both unigenes (Supplementary Figure 1A) and transcripts (Supplementary Figure 1B). Similar results were reported for dicots, including *Arabidopsis* (42.5%), soybean (*Glycine max*) (40.9%), carrot (*Daucus carota*) (41.03%), atish (*Aconitum heterophyllum)* (42%), and chickpea (*Cicer arietinum*) (40.3%) (Wang G. et al., 2015); but lower than monocots such as rice (*Oryza sativa*) (55%) and citronella *(Citronella winterianus)* (53.1%) (Carels and Bernardi, 2000). The numbers of unigenes decreased with an increase in the size of unigenes (Supplementary Figure 1C). The size distributions of unigenes indicate that most unigenes may not be predicted, indeed, 61.72% of 65,578 unigenes are noncoding sequences, some of which may be untranslated regions, noncoding RNAs, and intergenic spacers (Liu et al., 2012), only 38.28% had coding regions, and 15.67% had complete CDS (Coding sequence) (Supplementary Table 3).

**Table 1:** Statistics of *de novo* assembly of *Tetrastigma hemsleyanum* tuberous-root transcriptome.

**Item**	**All (number)**	**GC content (%)**	**Minimal length (bp)**	**Median length (bp)**	**Max length (bp)**	**Total assembled bases**	**N50**
Tanscripts	144,096	42.18	201	552.00	15,641	137,174,992	1,627
Unigenes	65,578	42.68	201	393.00	15,641	51,610,405	1,450

### Annotation of the Unigenes

The unigenes were aligned with the sequences available in six public databases, such as NR, GO, SwissProt, KEGG, eggNOG, and Pfam, with a cutoff value of *e* < 1 e^−5^. Of the 65,578 unigenes, 62.49% were matched to the sequences in these six public databases (Table 2, Supplementary Table 4), eggNOG (57.79%), NR (55.65%), GO (47.76%), Pfam (45.01%), SwissProt (37.08%), and KEGG (25.60%). Due to the lack of genomic and transcriptomic resources in SYQ, 37.51% of the unigenes could not be annotated to any of the searched databases, indicating that those unannotated sequences may be non-coding sequences (Liu et al., 2012) or unique to *T*. *hemsleyanum*. When blasted against the NR database by using BLASTx, 74.23% of the unigenes matched to the sequences of *Vitis vinifera*, followed by *Anthurium amnicola, Theobroma cacao*, barley, and other species (Supplementary Figure 1D), the observed high-level matching of SYQ and grapes was expected because both species belong to the same family Vitaceae, and are in near-evolutional relation with each other (Wang Y. H. et al., 2015). Similar results were observed in two lineages of *T*. *hemsleyanum* Central-South-East (*CSE*) 92.44% and Southwest (*SW*) 89.62%, separately (Wang et al., 2018). These two lineages are classified by using the chloroplast DNA (Wang Y. H. et al., 2015). However, CDS was available only for 25,106 unigenes (Supplementary Table 3), which is only about 50% of *T*. *hemsleyanum* lineages *CSE* (49,915) and *SW lineage (*54,373), the RNA-Seq of both *CSE* and *SW* lineages used leaf samples (Wang et al., 2018) and were also lower than *V. vinifera* genome, which has 30,434 protein-coding genes (Jaillon et al., 2007). Our finding suggests that the number of protein-coding genes discovered in the FD-TRs transcriptome is low. The number of active genes in FD-TRs may be lower than other tissues, for example, leaves and organs with fast growth rates in *T*. *hemsleyanum* plants. Therefore, a transcriptome analysis of different tissues of *T*. *hemsleyanum* at different stages of development may be necessary for better transcriptome profiling.

**Table 2:** Statistics of annotated unigenes from six databases.

**Protein database**	**Number of**	**Percent (%)**
	**unigenes**	
eggNOG	37,895	57.79
NR	36,494	55.65
GO	31,318	47.76
Pfam	29,516	45.01
Swissprot	24,319	37.08
KEGG	16,791	25.60
Total annotated unigenes	40,949	62.49
Total no of annotated unigenes	24,629	37.51
Total unigenes	65,578	100

The eggNOG database is used for exploring the ancestry of a protein, and to predict and classify unigenes. A total of 37,951 unigenes matched to the eggNOG protein database and were clustered into 23 categories (Supplementary Figure 2A). The largest category was “function unknown” (S, 17,760), followed by protein modification (O, 2,589), DNA (L, 2,114), transcription (K, 2,080), signaling (T, 1,937), translation (J, 1,718), carbohydrate (G, 1,436), etc., suggesting that most of the eggNOG annotated unigenes are mainly involved in basic life processes.

A total of 31,318 unigenes (Table 2) were assigned to 178,294 GO terms (Supplementary Figure 2B), of which 58,081, 65,663, and 54,550 terms were categorized into cellular components, biological processes, molecular functions. Among the cellular components, nucleus (7,870) and cytoplasm (5,474) received an ample representation followed by an integral component of membrane (33.3%), plasma membrane (12.1%), etc. Among the biological process, 39.7 and 36.5% regulates transcription and DNA-templated, which were the most represented process, followed by an oxidation-reduction process (14.7%), transcription, DNA-templated (12.5%), protein phosphorylation (10.6%), etc., while in the molecular function category, GO terms related to molecular function (37.4%) and protein binding (34.7%) were the most abundant functions, followed by ATP binding (6.0%) and RNA binding (5.7%).

**Figure 2:**
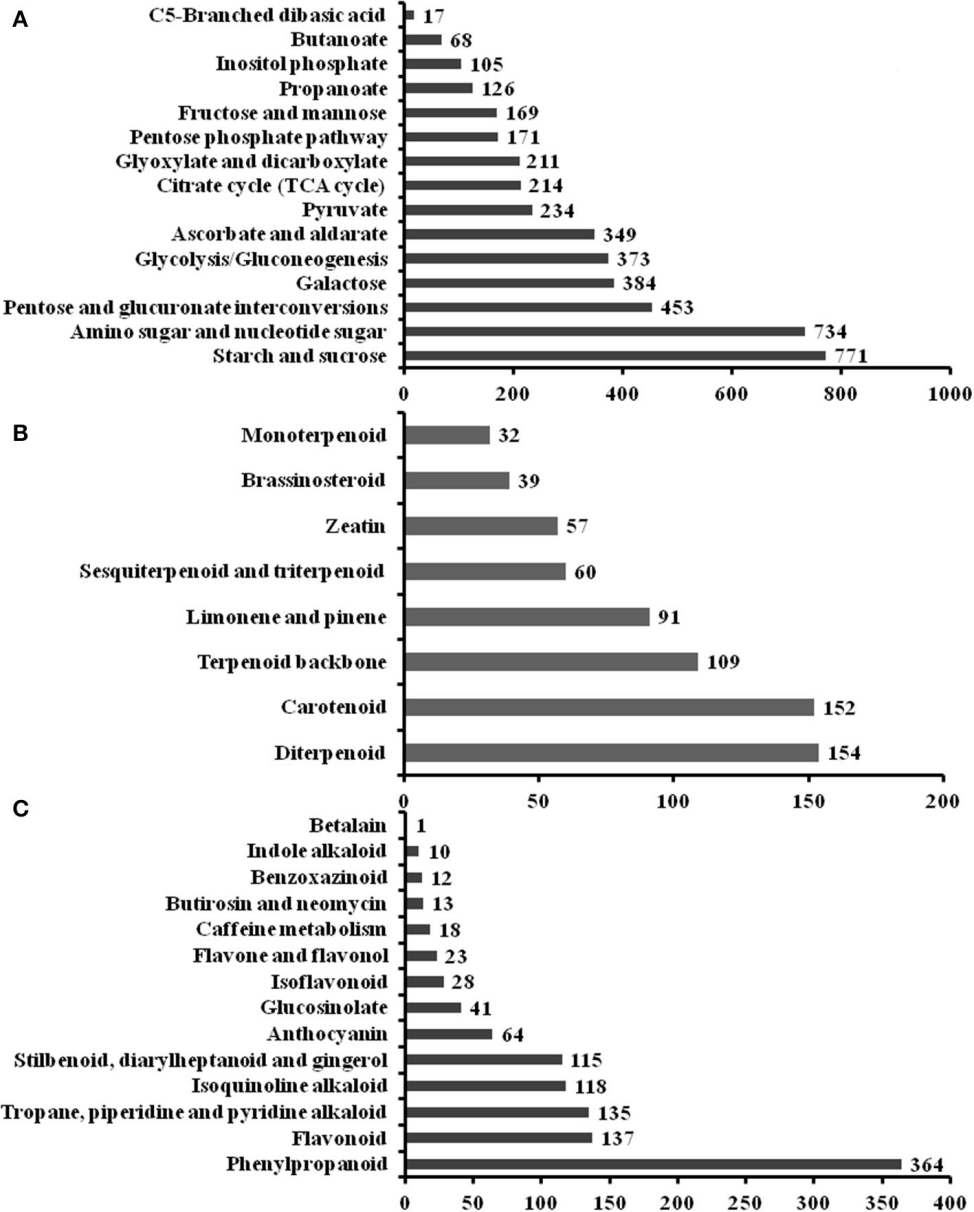
Summary of the Kyoto Encyclopedia of Genes and Genomes (KEGG) annotation of unigenes in sub-pathways of metabolism in *T. hemsleyanum* FD-TRs. **(A)** Carbohydrate metabolism, **(B)** metabolism of terpenoids and polyketides, and **(C)** biosynthesis of other secondary metabolites.

To elucidate active biosynthesis and metabolic pathways, we annotated the NR data with the KEGG database and identified 16,791 unigenes comprising 26,864 unique KO (KEGG Orthology) hits. The hits were assigned to the five main categories representing 19 sub-clusters. The highest number of KO identifiers was in metabolism (11,154), followed by genetic information processing (6,377), environmental information processing (1,510), organismal systems (1,493), and cellular processes (1,461). Pathways with the largest number of KO identifiers were carbohydrate metabolism (2,505), translation (2,448), folding, sorting, and degradation (2,260), environmental adaptation (1,493), transport and catabolism (1,461), etc. (Supplementary Figure 2C). Our analysis identified a large number of genes predicted to be involved in the biosynthesis of other secondary metabolites (898) and the metabolism of terpenoids and polyketides (702). For the metabolism of carbohydrates, the sub-pathway of starch and sucrose metabolism (771 unigenes) was the most highly represented metabolite (Figure 2A). For the metabolism of terpenoids and polyketides, the sub-pathways of diterpenoid metabolism (154), carotenoid metabolism (152), and terpenoid backbone metabolism (109) were the most represented metabolites (Figure 2B). Phenylpropanoid (364) and flavonoid (137) biosynthesis was the most represented sub-pathway of metabolism of other secondary metabolites (Figure 2C). These findings indicate that carbohydrate metabolism, phenylpropanoid and flavonoid biosynthesis, signal transduction factors may play key roles in the modulation of FD-TR developments responding to different seasons in *T*. *hemsleyanum*.

### Analysis of Gene Expression

We initially estimated the standardized expression data of unigenes based on FPKM values. To show the differences in the transcriptomic expression among the samples, all the standardized data were subjected to a Pearson correlation analysis and a principal component analysis (PCA), separately. Gene expression between the samples showed high correlations within the three repeats of most samples (Supplementary Figure 3): Sp (*R* is from 0.901 to 0.925), Su (0.838–0.951), Au (0.893–0.932), and Wi (0.917–0.981). The 12 samples were grouped into three with PC1 (51.97%) and PC2 (23.91%): group 1, Sp; group 2, Su and Au; and group 3, Wi (Supplementary Figure 4), indicating that the gene expression patterns in Su and Au have high similarity as compared to Sp and Wi, and high temperature in Au (29°C) and Su (37°C) might have activated more genes in *T*. *hemsleyanum* FD-TRs.

**Figure 3:**
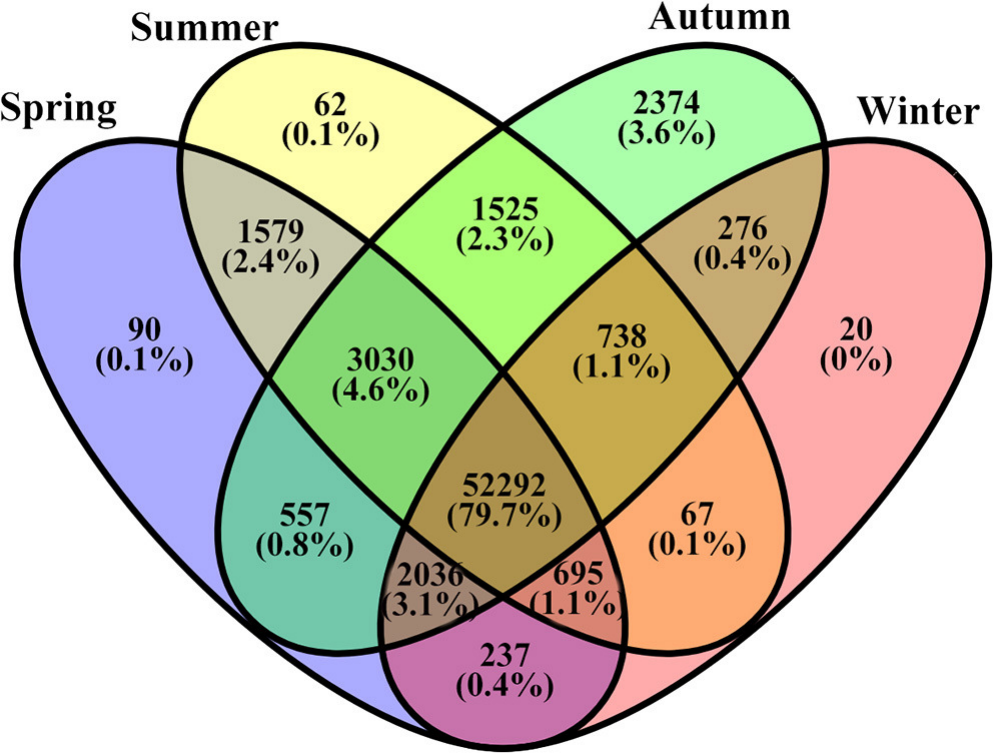
A Venn diagram of unigenes commonly and uniquely distributed in different seasonal FD-TRs in *T. hemsleyanum*.

The number of unigenes commonly or uniquely expressed in the samples from the four seasons is presented in Figure 3. Most unigenes (79.7%) were detected in all samples. About 4.6% unigenes were commonly expressed in the samples from the three seasons (Sp, Su, and Au), and 3.1% were common in the three seasons (Sp, Au, and Wi). Our findings also revealed uniquely detected unigenes: 90 in Sp samples, 20 in Wi. And surprisingly, 2,374 unigenes were exclusively detected in Au than in Su (62), although Su and Au were grouped together (Supplementary Figure 4). These results indicate that more unigenes may uniquely be activated in Au FD-TRs for the growth and accumulation of some storage resources for responding to low temperature in Wi and for the growth in next Sp.

### Analysis of DEGs

**Figure 4:**
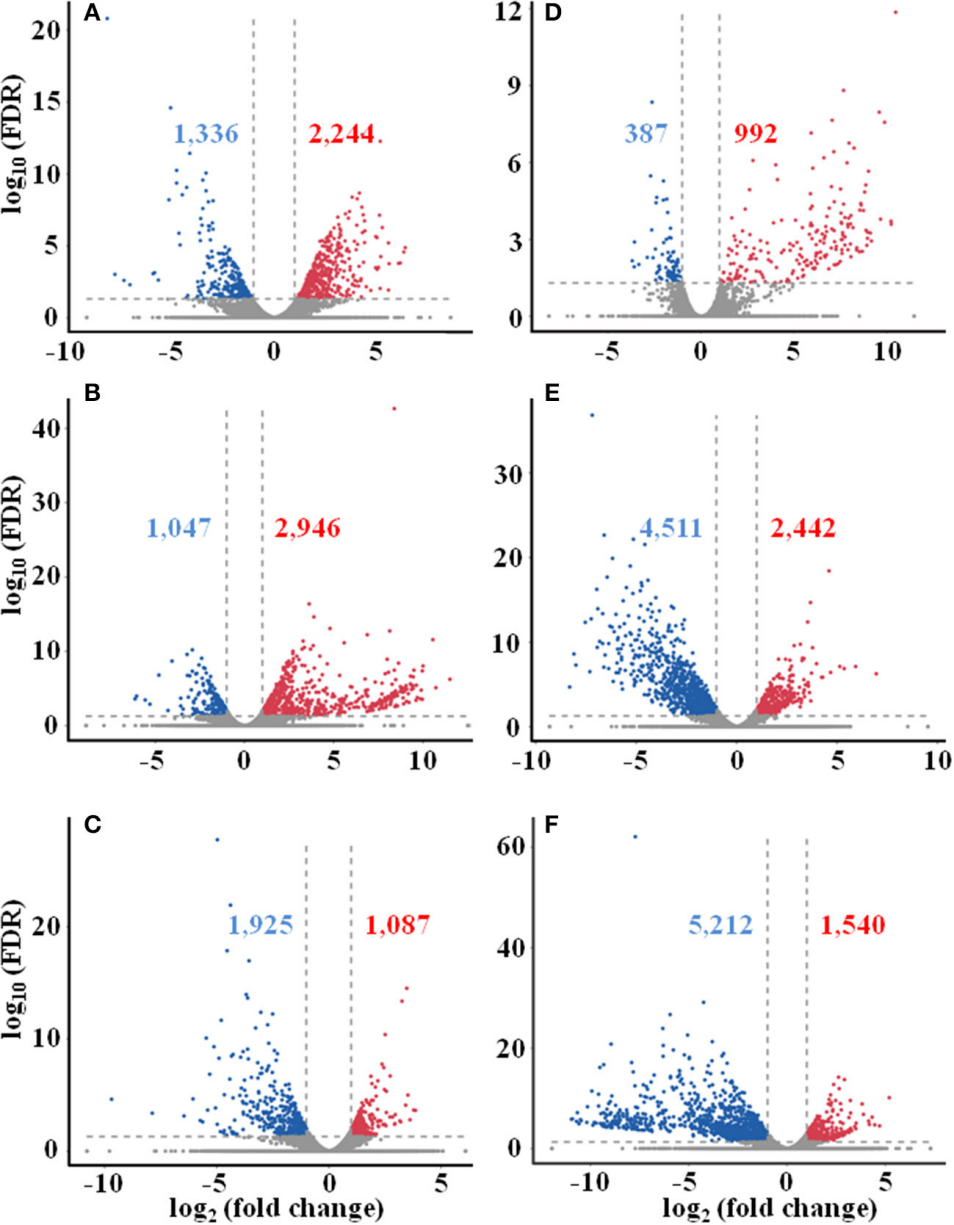
Distribution of differentially expressed unigenes in different seasonal FD-TRs in *T. hemsleyanum* using Volcano plot. **(A)** Su vs. Sp, **(B)** Au vs. Sp, **(C)** Wi vs. Sp, **(D)** Au vs. Su, **(E)** Wi vs. Su, and **(F)** Wi vs. Au. Red numbers and dots represent upregulated unigenes, blue number and dots represent downregulated unigenes, and gray dots represent unigenes with no change between comparing pairs. Sp, spring; Su, summer; Au, autumn; Wi, winter.

**Figure 5:**
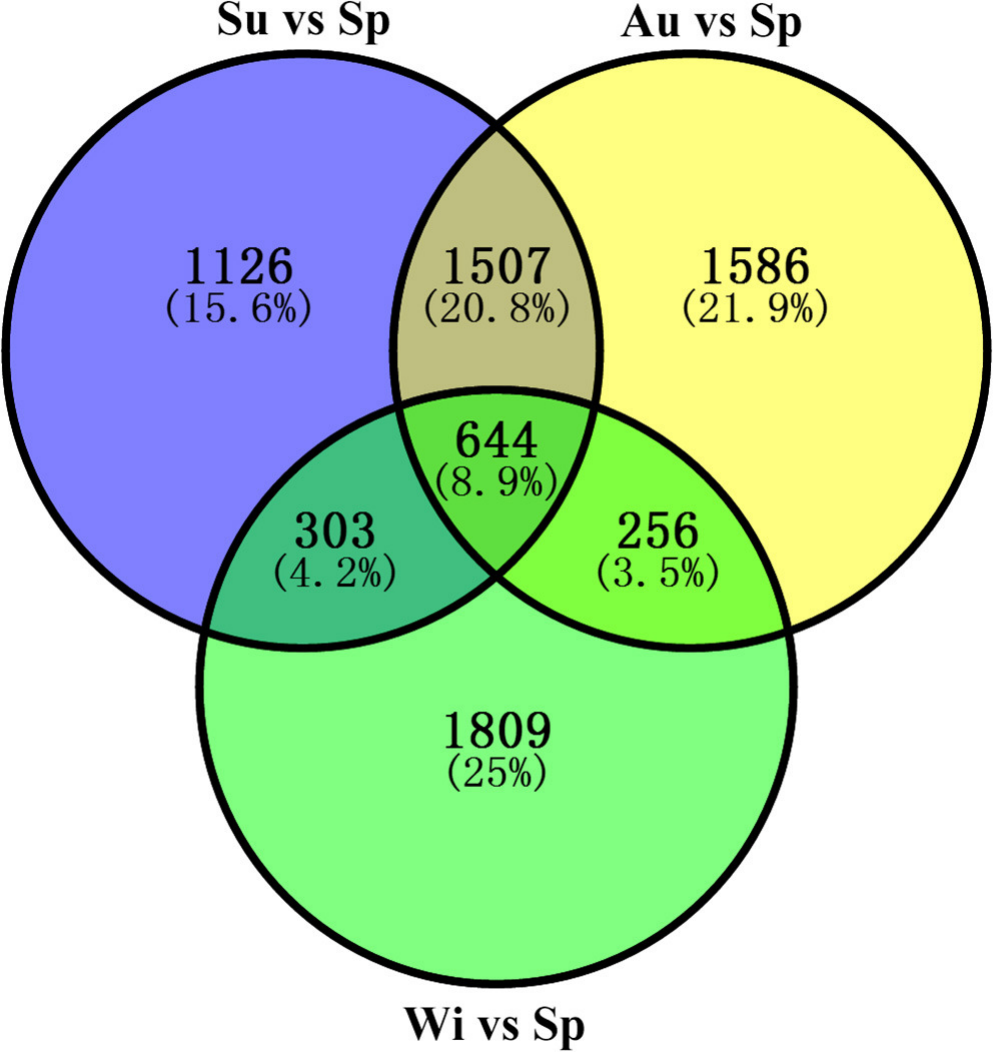
A Venn diagram showing the distribution of differentially expressed genes (DEGs) in Su, Au, and Wi samples by comparing with the Sp sample in *T. hemsleyanum* FD-TRs.

Differentially expressed genes (DEGs) were determined based on the transcript FPKM with |log_2_ FC| ≥ 1 and *p* < 0.05. Pair-wise comparison of transcripts between the samples of different seasons showed 3,580 DEGs in Su vs. Sp (2,244 upregulated and 1,336 downregulated), 3,993 in Au vs. Sp (2,946 and 1,047), 3,012 in Wi vs. Sp (1,087 and 1,925), 1,379 in Au vs. Su (992 and 387), 6,953 in Wi vs. Su (2,442 and 4,511), and 6,752 in Wi vs. Au (1,540 and 5,212) (Figure 4). These results indicate that most genes are silenced in Wi and Sp, but many genes were upregulated in Su and Au samples under higher temperatures. As shown in Figure 5, the number of common DEGs in Su, Au, and Wi compared to Sp was only about 8.9%, 25% genes were specific for Wi, following 21.9% for Au, and 15.6% for Su (Figure 5), indicating that most DEGs are specific to seasons. Interestingly, 20.8% DEGs were common for both Su vs. Sp and Au vs. Sp, suggesting that these DEGs have similar expression patterns, and could be a reason for Su and Au samples falling in the same group as shown in Supplementary Figure 4. The difference between Su and Au was the expression levels of some genes (Supplementary Table 4) due to a difference in the temperature during sampling days in Su and Au.

DEGs were annotated in the KEGG database, and were enriched into more than 116 pathways by using a hypergeometric test, of which more than 15 showed a significant enrichment and the top 20 pathways were shown in Table 3. The lowest number of pathways was in the group Au vs. Su, indicating that many DEGs have the same expression pattern in Au and Su samples; this is also supported by the PCA results (Supplementary Figure 4). The most highly enriched pathways were plant–pathogen interaction, plant hormone signal transduction, starch and sucrose metabolism, followed by amino sugar and nucleotide sugar metabolism, protein processing in endoplasmic reticulum, endocytosis, carbon metabolism, phenylpropanoid biosynthesis, etc. With the exception of a plant–pathogen interaction group, many genes in phytohormone signaling and starch and sucrose metabolism were activated in different seasons in *T*. *hemsleyanum* FD-TRs.

**Table 3:** Kyoto encyclopedia of genes and genomes (KEGG) enrichment of differentially expressed genes (DEGs) with 20 top categories (*q* < 0.05).

**Code**	**Pathway entry**	**Pathway definition**	**DEGs (significant enrichment pathways/total matched pathways)**						**Total**
			**Su vs. Sp**	**Au vs. Sp**	**Wi vs. Sp**	**Au vs. Su**	**Wi vs. Su**	**Wi vs. Au**	
			**(31/132)**	**(30/130)**	**(24/128)**	**(15/116)**	**(24/135)**	**(30/133)**	
1	map04626	Plant-pathogen interaction	204	219	107	39	351	319	1,239
2	map04075	Plant hormone signal transduction	159	155	94	30	246	210	894
3	map04141	Protein processing in endoplasmic reticulum	91	73	67	54	144	154	583
4	map00500	Starch and sucrose metabolism	119	109	95	50	186	166	725
5	map00520	Amino sugar and nucleotide sugar metabolism	92	107	48	37	180	174	638
6	map04144	Endocytosis	88	81	44	33	161	150	557
7	map00940	Phenylpropanoid biosynthesis	63	69	53	24	109	103	421
8	map00010	Glycolysis/Gluconeogenesis	52	52	30	21	61	75	291
9	map00710	Carbon fixation in photosynthetic organisms	30	40	24	14	42	52	202
10	map01200	Carbon metabolism	74	92	51	36	106	139	498
11	map04712	Circadian rhythm–plant	40	32	27	7	55	50	211
12	map04145	Phagosome	23	25	23	16	36	60	183
13	map00941	Flavonoid biosynthesis	39	39	31	15	61	50	235
14	map00360	Phenylalanine metabolism	31	34	20	4	54	57	200
15	map00592	Alpha-Linolenic acid metabolism	29	34	18	7	37	40	165
16	map00051	Fructose and mannose metabolism	29	26	20	9	34	36	154
17	map00410	Beta-Alanine metabolism	25	28	20	6	37	47	163
18	map00040	Pentose and glucuronate interconversions	39	35	33	25	90	82	304
19	map00945	Stilbenoid, diarylheptanoid, and gingerol biosynthesis	18	28	19	11	42	40	158
20	map00350	Tyrosine metabolism	27	34	27	8	49	53	198
Total			1,530	1,595	1,068	545	2,488	2,503	9,729

*Su, summer; Sp, spring; Au, autumn; Wi, winter*.

Therefore, we next focused on three groups of genes that may be involved in key pathways of FP biosynthesis, starch and sucrose metabolism, and phytohormone signaling. FPs are the main components in *T*. *hemsleyanum* TRs for medicine (Guo et al., 2019), and starch and sucrose metabolism and phytohormone signaling were enriched significantly based on DEGs (Table 3).

### Unigenes in FP Biosynthesis Pathways

**Figure 6:**
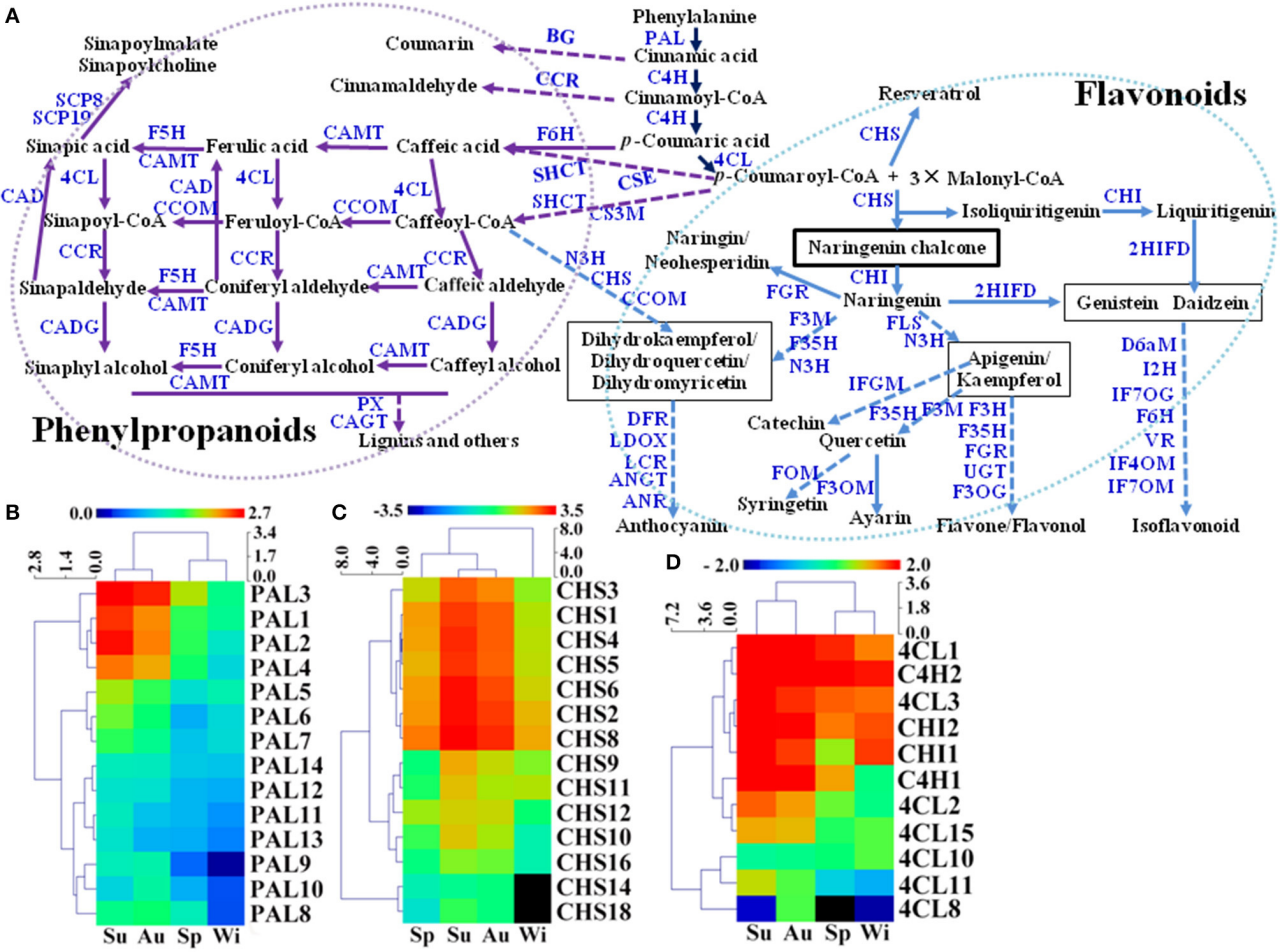
Putative biosynthetic pathways of flavonoids and phenylpropanoids (FPs) (referring to KEGG pathways map00940 and map00941) in *T. hemsleyanum* FD-TRs **(A)** and heatmaps representing expression dynamics of genes involved in phenylalanine ammonia-lyases [PAL, **(B)**], chalcone synthases [CHS, **(C)**], 4-coumarate-CoA ligases (4CL), trans-cinnamate 4-monooxygenases (C4H), and chalcone isomerase [CHI, **(D)**]. 4CL, 4-coumarate-CoA ligase; 2HIFD, 2-hydroxyisoflavanone dehydratase; ANGT, anthocyanidin 5,3-O-glucosyltransferase; ANR, anthocyanidin reductase; BG, beta-glucosidase; C4H, trans-cinnamate 4-monooxygenase; CAD, coniferyl-aldehyde dehydrogenase; CADG, cinnamyl-alcohol dehydrogenase; CAGT, coniferyl-alcohol glucosyltransferase; CAMT, caffeic acid 3-O-methyltransferase; CCOM, caffeoyl-CoA O-methyltransferase; CCR, cinnamoyl-CoA reductase; CHI, chalcone isomerase; CHS, chalcone synthase; CS3M, coumaroylquinate (coumaroylshikimate) 3′-monooxygenase; CSE, caffeoylshikimate esterase; D6aM, 3,9-dihydroxypterocarpan 6a-monooxygenase; DFR, dihydroflavonol-4-reductase; F3M, flavonoid 3′-monooxygenase; F3OG, flavonol 3-O-glucosyltransferase; F3OM, flavonol 3-O-methyltransferase; F35H, flavonoid 3′, 5′-hydroxylase; F5H, ferulate-5-hydroxylase; F6H, flavonoid 6-hydroxylase; FGR, flavanone 7-O-glucoside 2″-O-beta-L-rhamnosyltransferase; FLS, flavonol synthase; FOM, flavonoid O-methyltransferase; I2H, isoflavone 2′-hydroxylase; IF7OG, isoflavone 7-O-glucosyltransferase; IF7OM, isoflavone-7-O-methyltransferase; IF4OM, isoflavone 4-O-methyltransferase; IFGM, isoflavone 7-O-glucoside-6′-O-malonyltransferase; N3H, naringenin 3-dioxygenase; LCR, leucoanthocyanidin reductase; LDOX, leucoanthocyanidin dioxygenase; PAL, phenylalanine ammonia-lyase; PX, peroxidase; SCP8, serine carboxypeptidase-like 8; SCP19, serine carboxypeptidase-like 19; SHCT, shikimate O-hydroxycinnamoyltransferase; UGT, UDP-glucosyl transferase; VR, vestitone reductase.

Flavonoids and phenylpropanoids are the two groups of the most abundant secondary metabolites in *T*. *hemsleyanum* TRs for their therapeutic properties on anticancer, anti-inflammatory, and antiviral properties (Guo et al., 2019). In addition, secondary metabolites including FPs may play pivotal roles in plant development and tolerance to biotic and abiotic stresses such as extreme temperature, drought, UV, nutrient deficiencies, pathogens, and herbivores (Isah, 2019), but little is known about their biosynthetic pathways in SYQ TRs. A total of 554 unigenes were predicted to be involved in biosyntheses of FPs in SYQ FD-TRs, and 255 DEGs were detected (Figure 6A, Supplementary Table 5). FP biosynthesis pathways use the same upstream steps that produce *p*-coumaroyl-CoA, which is synthesized from L-phenylalanine by using enzymes such as phenylalanine ammonia-lyase (PAL), cinnamate 4-hydroxylase (C4H), and 4-coumarate-CoA ligase (4CL). Phenylalanine is the precursor of phenylpropanoids, flavonoids, and other aromatic compounds. The expression of 14 PAL, 2 C4H, and 7 out of 18 4CL unigenes forms DEG between FD-TRs samples of the four seasons (Figure 6A). Most PAL genes were highly expressed in the Su samples (especially PAL1, 2, 3, and 4), followed by Au, Sp, and Wi (Figure 6B); C4H2 was highly expressed in the samples of all the four seasons; C4H1 was mainly expressed in Su and Au; and 4CL1, 2, and 3 were more abundantly expressed in Su, Au, and Sp (Figure 6D). These results indicate that the genes upstream of FPs are more activated in Su and Au than in Sp and Wi.

Intermediates of flavonoid biosynthesis, naringenin, or liquiritigenin are biosynthesized from *p*-coumaroyl-CoA by chalcone synthase (CHS) and chalcone isomerase (CHI). As shown in Figure 6C, 14 of the 18 CHS unigenes were DEGs between Su and Au samples; and both two CHIs were expressed at lower levels in Sp than in Su, Au, and Wi samples (Figure 6D). A previous study showed that 37 CHS and 5 CHI unigenes were identified in young SYQ leaves, of which some were shown to be activated by short-term cold treatment (48 h) (Peng et al., 2019), suggesting that there are more CHSs and CHIs activated in young leaves exposed to transient cold treatment than in FD-TRs under long-term cold stress in Wi.

The downstream pathway of flavonoid biosynthesis is divided into three branches of biosynthesis: flavone/flavonol, anthocyanidin, and isoflavonoid. Putative flavone/flavonol biosynthesis may be catalyzed by naringenin 3-dioxygenase (N3H) and flavonol synthase (FLS) to synthesize apigenin or kaempferol, then undergoing further modification by methyltransferases, hydroxylases, glucosyltransferases, and rhamnosyltransferases to form flavones or flavonols (Figure 6A). During anthocyanidin biosynthesis, the formation of three compounds (dihydrokaempferol, dihydroquercetin, and dihydromyricetin) from naringenin was made by using enzymes such as N3H, flavonoid 3′, 5′-hydroxylase (F35H), and flavonoid 3′-monooxygenase (F3M). Dihydrokaempferol may also be synthesized from caffeoyl-CoA by using enzymes such as N3H, CHS, and caffeoyl-CoA O-methyltransferase (CCOM). These three molecules are modified by reductases, dioxygenases, and glucosyltransferases to form anthocyanidin (Figure 6A). For the isoflavonoid biosynthesis, genistein or daidzein may be formed of 2-hydroxyisoflavanone dehydratase (2HIFD) and CHI, and then genistein or daidzein was catalyzed by methyltransferases, monooxygenases, hydroxylases, glucosyltransferases, and reductase to synthesize isoflavonoids (Figure 6A).

Phenylpropanoids are a large and complex group of secondary metabolites in plants. In SYQ FD-TRs, the main pathway in the biosynthesis of phenylpropanoids may be similar to other plants. Caffeic acid and caffeoyl-CoA are synthesized from *p*-coumaroyl-CoA and *p*-coumaric acid by flavonoid 6-hydroxylase (F6H), shikimate O-hydroxycinnamoyltransferase (SHCT), coumaroylshikimate 3′-monooxygenase (CS3M), and caffeoylshikimate esterase (CSE). They are then catalyzed by 4CL, CCOM, cinnamoyl-CoA reductase (CCR), cinnamyl-alcohol dehydrogenase (CADG), caffeic acid 3-O-methyltransferase (CAMT), and other enzymes to form lignin and related compounds (Figure 6A). Therefore, these findings may offer some valuable genomic resources for engineering the biosynthesis pathways of FPs in *T*. *hemsleyanum*.

### Contents of FPs in FD-TRs

The expression levels of most genes involved in the FP biosynthesis pathways were upregulated in Su and Au (Figure 6A). Thus, the total flavonoids and some components of FPs were detected to analyze their possible correlations with gene activities in their biosynthesis pathways. Significant differences (*p* < 0.05) were observed among the samples of four seasons. The highest amount (18.848 mg/g) and the lowest (7.369 mg/g) was observed in Au and Wi samples, separately, and the contents of total flavonoids increased from Sp to Au but decreased in Wi (Supplementary Figure 5A). Enhanced flavonoid content observed in Au coincided with the harvest season when TRs have the highest nutrient levels such as starch and secondary metabolites.

**Figure 7:**
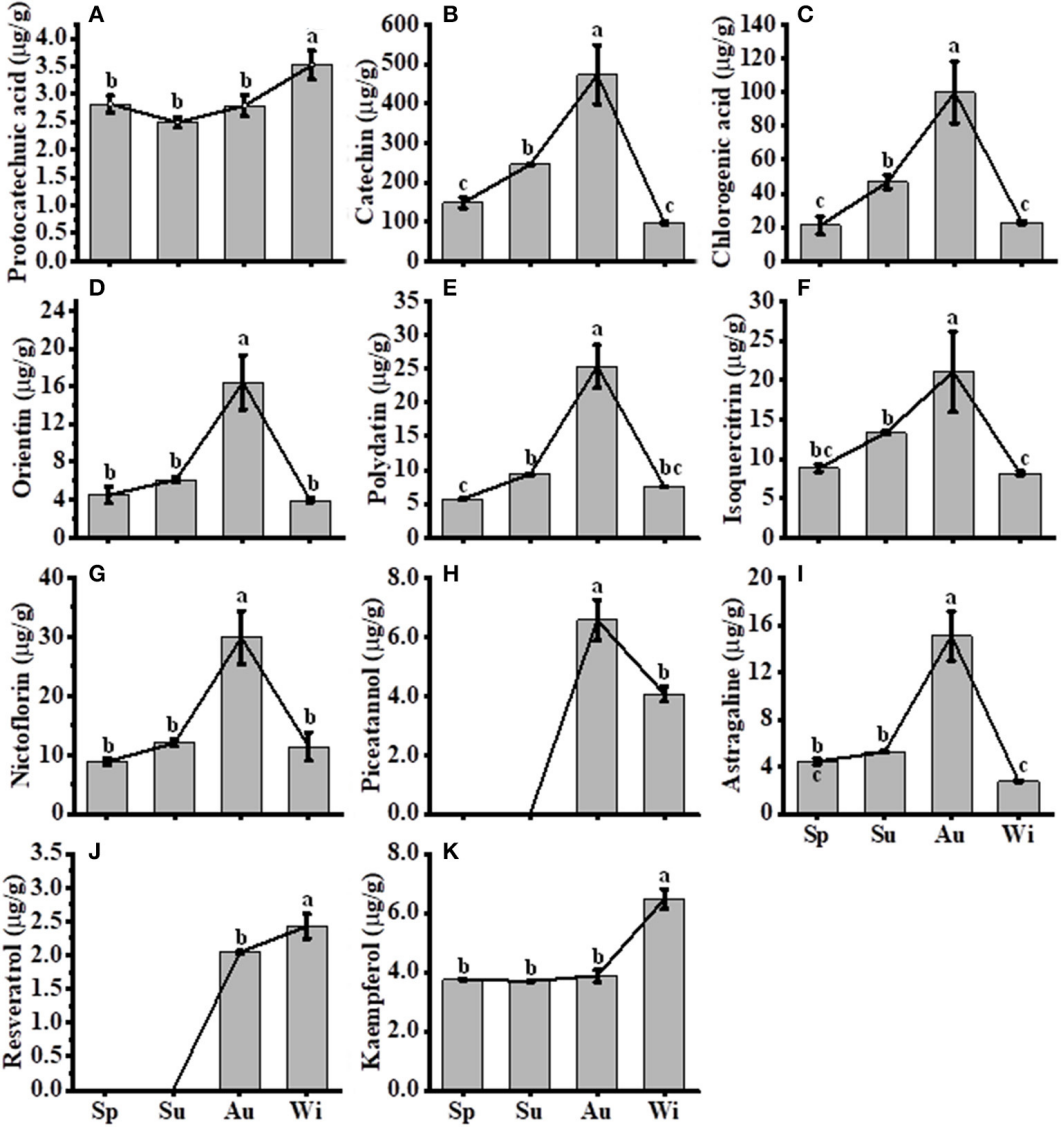
Contents of compounds in *T. hemsleyanum* FD-TRs. Sp, Spring; Su, Summer; Au, Autumn; Wi, Winter. **(A)** protocatechuic acid, **(B)** catechin, **(C)** chlorogenic acid, **(D)** orientin, **(E)** polydatin, **(F)** isoquercitrin, **(G)** nictoflorin, **(H)** piceatannol; **(I)** astragaline; **(J)** resveratrol, and **(K)** kaempferol. Bars represent means + SE (*n* = 5). Bars bearing the same letter are not significantly different.

A total of 11 FPs were separated and identified by using HPLC (Supplementary Figure 5F), where nine compounds, including protocatechuic acid, catechin, chlorogenic acid, orientin, polydatin, isoquercitrin, nictoflorin, astragaline, and kaempferol, out of 11 FPs were detected in all samples. Two compounds piceatannol and resveratrol were detected in Au and Wi samples only, but three compounds including myricetin, quercetin, and naringenin were nearly unidentified in all samples (Supplementary Figures 5B–E). The level of seven FPs, including catechin (Figure 7B), chlorogenic acid (Figure 7C), orientin (Figure 7D), polydatin (Figure 7E), isoquercitrin (Figure 7F), nictoflorin (Figure 7G), and astragaline (Figure 7I), increased from Sp to Au and decreased in Wi. These results suggest that FPs may be degraded into other components in response to a prolonged lower temperature condition in Wi as compared to samples from other seasons. The contents of protocatechuic acid (Figure 7A) and kaempferol (Figure 7K) were higher in Wi. Piceatannol (Figure 7H) and resveratrol (Figure 7J) were only detected in Au and Wi. Piceatannol was accumulated at higher levels in Au than in Wi (Figure 7H), but resveratrol had the contrary results (Figure 7J), indicating that a seasonal variation in the type of compounds accumulated in FD-TR may affect its medicinal efficacy and needs further studies. Total flavonoids and some FP compounds were accumulated at higher levels in Au and Su, which coincided with an enhanced expression of some genes, such as PALs (Figure 6B), CHSs (Figure 6C), C4H1s, 4CL1s, and CHIs, in the FP biosynthesis pathways (Figure 6D). The contents of total flavonols and some flavonol intermediates were also correlated with the expression level of flavonol synthesis-related genes *CHS* and *CHI* in SYQ young leaves under cold conditions (Peng et al., 2019), and tobacco and *Arabidopsis* plants under chilling stress (Zhang et al., 2011; Meng et al., 2015). In addition, some flavonoids may play a role under cold conditions, for example, a higher flavonoid content in transgenic tobacco plants overexpressing *SlF3HL* (coding flavonone 3-hydroxylase-like protein), a flavonoid biosynthesis gene, reduced contents of malondialdehyde, H_2_O_2_, and ${\text{O}}_{2}^{-}$ under chilling stress (Meng et al., 2015), suggesting their role of antioxidation. These results indicate that some flavonoid biosynthesis genes and flavonoid metabolites may transiently be enhanced by short-term cold treatment, decreased with long-term low temperature in Wi, or may have different roles in specific tissues of organs including leaf and root. The roles of flavonoid biosynthesis genes and flavonoid metabolites in SYQ FD-TRs responding to a long-term cold condition need to be studied further.

### Unigenes Related to Starch and Sucrose Metabolism

**Figure 8:**
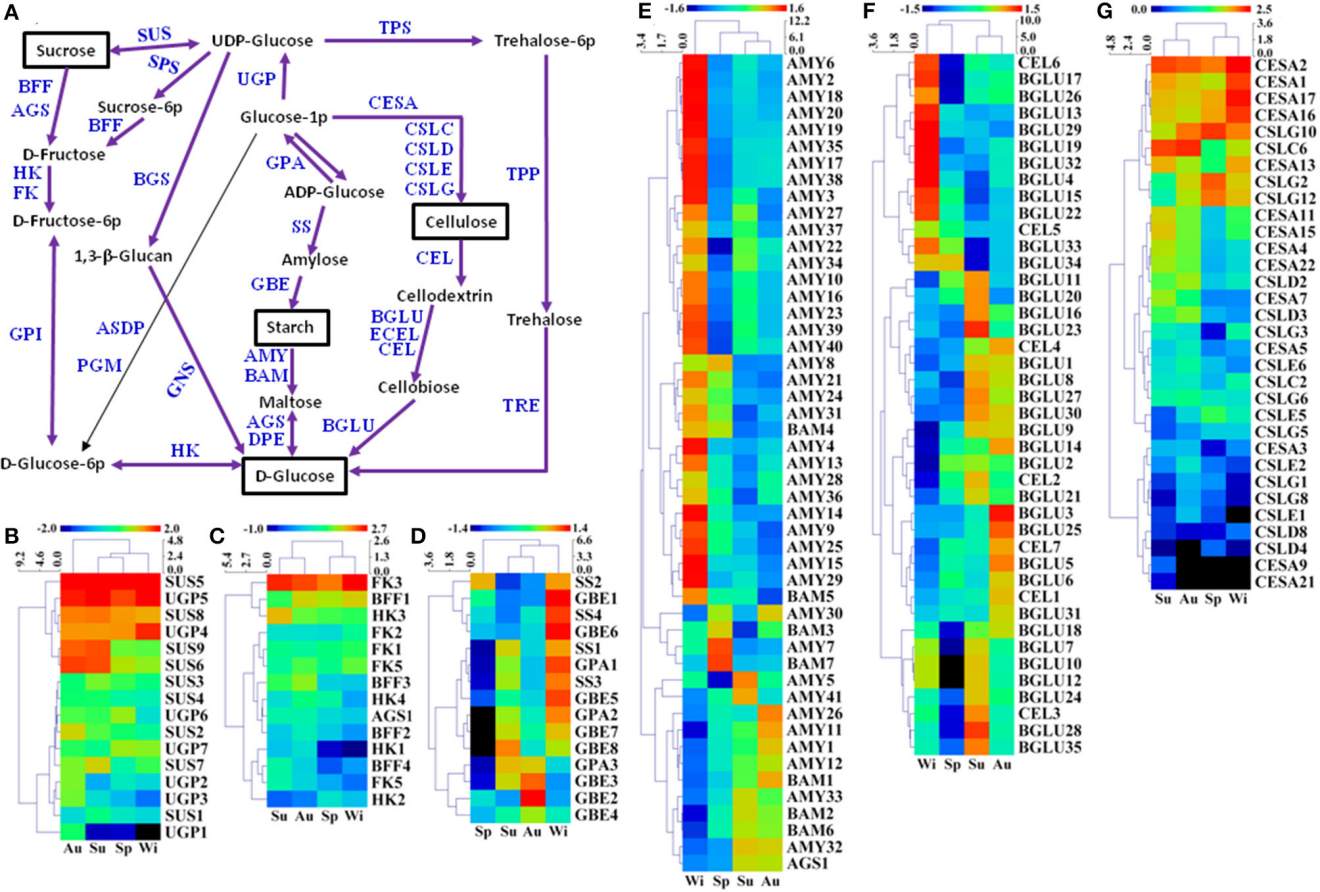
A putative model of starch and sucrose metabolism (referring to the KEGG pathway map00500) in *T. hemsleyanum* FD-TRs **(A)** and heatmaps representing expression dynamics of genes involved in sucrose biosynthesis **(B)**, sucrose degradation **(C)**, starch biosynthesis **(D)**, starch degradation **(E)**, cellulose degradation **(F)**, and cellulose biosynthesis **(G)**. AGS, α-glucosidase; AMY, α-amylase; ASDP, ADP-sugar diphosphatase; BAM, beta-amylase; BFF, beta-fructofuranosidase; BGLU, beta-glucosidase; BGS, 1,3-beta-glucan synthase; CEL, endoglucanase; CESA, cellulose synthase A; CSLC-CSLG, cellulose synthase-like protein C-G; DPE, 4-α-glucanotransferase; ECEL, cellulose 1,4-beta-cellobiosidase; FK, fructokinase; GBE, 1,4-α-glucan branching enzyme; GNS, glucan endo-1,3-beta-glucosidase; GPA, glucose-1-phosphate adenylyltransferase; GPI, glucose-6-phosphate isomerase; HK, hexokinase; PGM, phosphoglucomutase; SPS, sucrose-phosphate synthase; SS, starch synthase; SUS, sucrose synthase; TPP, trehalose 6-phosphate phosphatase; TPS, trehalose 6-phosphate synthase/phosphatase; TRE, α,α-trehalase; UGD, UDP-glucose 6-dehydrogenase; UGP, UTP-glucose-1-phosphate uridylyltransferase.

Starch and sucrose are essential in plant growth and development, which are involved in primary and secondary metabolic pathways, and also play key roles in the initiation and growth of TRs (Rohde et al., 2004). A total of 616 unigenes were predicted to be involved in starch and sucrose metabolism, and 213 of 616 unigenes were DEGs (Figure 8A, Supplementary Table 7). For sucrose biosynthesis, glucose-1-phosphate (Glucose-1P) is metabolized from ADP-glucose by glucose-1-phosphate adenylyltransferase (GPA). GPA1 and GPA2 were expressed at a higher level in Wi, but a lower in Sp samples while GPA3 was expressed at a higher level in Su and Au, but a lower level in Sp and Wi (Figure 8D). Glucose-1-P is then catalyzed to UDP-glucose by UTP-glucose-1-phosphate uridylyltransferase (UGP), and the final step from UDP-glucose to sucrose is metabolized by sucrose synthase (SUS). The expression level of SUS5, SUS8, UGP4, and UGP5 was high in all the samples; SUS6 and SUS9 were expressed at a higher level in Su and Au FD-TRs (Figure 8B), indicating that some sucrose-biosynthesis genes are activated in roots throughout the year while many sucrose-biosynthesis genes were upregulated only in the Su and Au, suggesting more carbon from source leaves is transferred into other metabolisms in Su and Au FD-TRs. Among these genes, SUS catalyzes the reversible conversion of sucrose and uridine diphosphate to fructose and UDP-glucose (Ruan, 2014), and is a key enzyme facilitating carbon allocation for metabolism or storage organs. Plants with mutation or RNA interference of *SUS* have been shown to reduce seed mass, starch content, fruit setting, and sucrose unloading, and disrupt the integration of endosperm cell wall and seed development (Xu et al., 2019). In addition, a high sucrose concentration has been implicated in *in vitro* TR induction (Tsubone et al., 2000). These results suggest that sucrose and its biosynthesis gene *SUS* play an important role in SYQ FD-TRs, and SUS4 and SUS1 are also upregulated in *C. speciosa* and *R. sativus* TRs (Mitsui et al., 2015; Xu et al., 2016). During catabolic reactions, sucrose is converted to D-fructose by beta-fructofuranosidase (BFF) and α-glucosidase (AGS), and then to D-fructose-6-P by hexokinase (HK) and fructokinase (FK) (Figure 8A). Interestingly, only FK3 was upregulated in Su, Au, and Wi while most of the genes involved in sucrose degradation were activated in Su and Au, except HK2, FK2, and FK5 (Figure 8C), also indicating that sucrose transported from the source leaves is transferred into other compounds in FD-TRs in Su and Au. In addition, as a signal transduction factor, sucrose is one of the driving forces for the formation and growth of TRs (Koch, 2004), and sucrose has also been reported to interact with other signal transduction factors to modulate TR architecture, biosynthesis, and perception of hormones, such as ABA (Rolland et al., 2002; Koch, 2004) and CTKs (Eguchi and Yoshida, 2008; Tanaka et al., 2008). These results also suggest that sucrose may play an important role of a signaling molecule in SYQ FD-TRs.

During starch biosynthesis, amylose is formed from ADP-glucose by SS, and the starch branching is catalyzed from amylose by 1,4-α-glucan branching enzyme (GBE). In this study, the expression of four SS, five GBE, and two GPA genes was higher in Wi than in other seasons and reached the lowest level in Sp while the expression of GPA3 and GBE2-4 was higher in Au (Figures 8A,D). Stored starch is generally degraded in the cold Wi (Chinnasamy and Bal, 2003), but many genes in the starch biosynthesis in FD-TRs were upregulated in Wi. For starch degradation, there were 165 unigenes coding α-amylases (AMYs), of which 41 were DEGs. Starch is degraded to maltose by AMYs or β-amylases (BAM). Maltose is then catalyzed to D-glucose by AGS and 4-α-glucanotransferases (DPE), but the expression of two DPE genes was not significantly different among the samples of four seasons (Figure 8E). Similar to starch biosynthesis, the expression of most unigenes involved in starch degradation (32 AMYs and 2 BAMs) were upregulated in Wi, only AMY7, AMY30, BAM3, and BAM7 were upregulated in Sp, and 8 AMYs, 3 BAMs, and AGS1 were expressed at higher levels in Su and Au (Figure 8E), indicating that AMYs, which are endo-acting starch degrading enzymes (Zeeman et al., 2010), may play an important role in starch degradation in Wi FD-TRs. Some genes implicated in starch and sucrose metabolism have been reported to be upregulated in TRs of other species, for example, genes encoding BAM, AGPase/GPA, GBE, and SS (Wang S. et al., 2006; Firon et al., 2013; Ponniah et al., 2017). *BAMs* and *AGPases* have been shown to be activated during TRs initiation (Firon et al., 2013; Saithong et al., 2013; Li et al., 2019), which are positively correlated with their roles in starch and sucrose biosynthesis (Ihemere et al., 2006; Saithong et al., 2013), and the increased expression of *AGPase* has been shown to enhance biomass yield in sweetpotato (Firon et al., 2013; Villordon et al., 2014) and cassava (Ihemere et al., 2006). Our findings indicate that starch metabolism is more complex in FD-TRs among seasons. Many genes in both starch biosynthesis (Figure 8D) and degradation (Figure 8E) pathways were activated in Wi, suggesting that the balance of intermediates of starch metabolism may be important for maintaining FD-TR and to respond to low temperatures in Wi.

Our findings also revealed 60 predicted unigenes as cellulose-biosynthetic genes, including 54 genes belonging to glycosyltransferase family, and 32 out of 54 glycosyltransferase family genes were DEGs (Figure 8A). Most of the 32 genes were significantly expressed in Su and Au (Figure 8G). Endoglucanases (CEL3), β-glucosidases (BGLU), and cellulose 1,4-β-cellobiosidase (ECEL) were responsible for the degradation of cellulose to D-glucose (Figure 8A). A total of 11 BGLUs and 2 CELs genes were expressed at higher levels in Wi than in other seasons, 14 and 3 CEL genes were highly expressed in Su, and 14 BGLUs and 2 CELs were highly expressed in Au. However, none of the CEL and BGLU genes was upregulated significantly in Sp (Figure 8F). Overall, 11 cellulose degradation genes (GLUs, Figure 8F) were activated in Wi, but only 3 cellulose biosynthesis genes (CESAs, Figure 8G) were activated in Wi. These results indicate that more cellulose might be degraded under extended conditions of low temperatures in Wi.

### Unigenes in Phytohormone Signal Transduction Pathways

**Figure 9:**
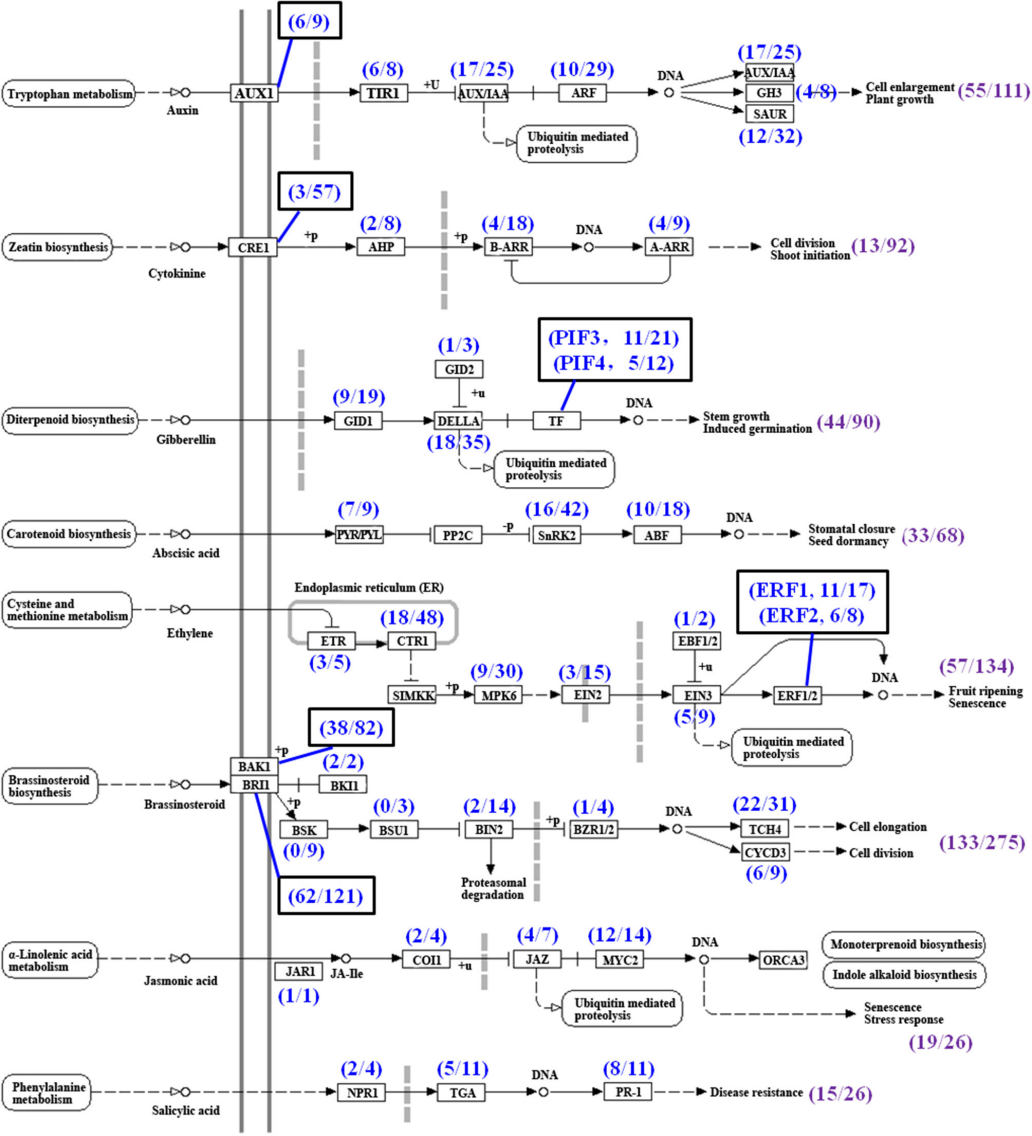
Summary of unigenes in phytohormone signal transduction pathways (referring to the KEGG pathway map04075) in *T. hemsleyanum* FD-TRs. AUX1, auxin influx carrier; TIR1, transport inhibitor response 1; AUX/IAA, auxin-responsive protein/indole-3-acetic acid; ARF, auxin response factor; GH3, auxin-responsive GH3 gene family; SAUR, small auxin-up RNA family protein; CRE, Arabidopsis histidine kinase 2/3/4 (cytokinin receptor); AHP, histidine-containing phosphotransfer protein; A-ARR, two-component response regulator A-ARR family; B-ARR, two-component response regulator B-ARR family; GID1, gibberellin receptor GID1; GID2, F-box protein GID2; PIF, phytochrome-interacting factor; PYL/PYL, abscisic acid receptor PYR/PYL family; SnRK2, serine/threonine-protein kinase SRK2; ABF, ABA-responsive element binding factor; ETR, ethylene receptor; CTR1, serine/threonine-protein kinase CTR1; MPK6, mitogen-activated protein kinase 6; EIN2, ethylene-insensitive protein 2; EIN3, ethylene-insensitive protein 3; EBF1/2, EIN3-binding F-box protein; ERF, ethylene-responsive transcription factor; BAK1, brassinosteroid insensitive 1-associated receptor kinase 1; BRI1, protein brassinosteroid insensitive 1; BKI1, BRI1 kinase inhibitor 1; BSK, BR-signaling kinase; BSU1, serine/threonine-protein phosphatase BSU1; BIN2, protein brassinosteroid insensitive 2; BZR1/2, brassinosteroid resistant 1/2; TCH4, xyloglucan:xyloglucosyl transferase TCH4; CYCD3, cyclin D3; JAR1, jasmonic acid-amino synthetase; COI-1, coronatine-insensitive protein 1; JAZ, jasmonate ZIM-domain-containing protein; MYC2, transcription factor MYC2; NPR1, regulatory protein NPR1; TGA, transcription factor TGA; PR-1, pathogenesis-related protein 1, Sp, spring; Su, summer; Au, autumn; Wi, winter.

Many phytohormones, including CTKs, auxin, BRs, JA, GA, ethylene, and ABA, and their signal transduction factors participate in the regulation of TR formation and development (Koda et al., 2001; Wang Q. et al., 2006; Noh et al., 2010; Sojikul et al., 2015; Ding et al., 2020). In this study, phytohormone signaling was the second most enriched group in the KEGG analysis by using a hypergeometric test (Table 3). A total of 822 unigenes possibly involved in eight phytohormone signaling were enriched (Figure 9, Supplementary Table 8) while the genes involved in the biosyntheses of some phytohormones were not enriched significantly (Figure 2B), indicating that the phytohormone signal transduction factors may modulate FD-TRs independent of the biosynthesis of phytohormones or the content of phytohormone plays a basal role in FD-TRs in the four seasons. Most signal transduction factors of hormones, excluding auxin, were upregulated in Su and Au (Supplementary Figure 6).

A total of 133 DEGs out of 275 putative unigenes belonged to the BR signal transduction factors, including BR insensitive 1 (BRI1), BRI-associated receptor kinase 1 (BAK), xyloglucan: xyloglucosyl transferase (TCH4), cyclin D3 (CYCD3), protein BR insensitive 2 (BIN2), BRI1 kinase inhibitor 1 (BKI1), and BR resistant 1/2 (BZR1/2), but the expression of nine of the BR-signaling kinase (BSK) and three of the BSU1 (serine/threonine-protein phosphatase BSU1) were not markedly different in FD-TRs samples of all seasons (Figure 9). BRs generally participate in the regulation of meristem size and xylem differentiation to control root growth and development (González-García et al., 2011; Nolan et al., 2017). BRs have been shown to promote the tuber number in potato and petiole elongation in carrot by altering contents of polyamine, GA, and cellulose deposition (Wei and Li, 2016; Que et al., 2018). Of the 133 DEGs, 23 genes in Sp, 29 in Su, 35 in Au, and 20 in Wi were expressed at higher levels, only CYCD3-9, BRI1-63, and BAK1-38 were commonly upregulated in Wi, Su, and Au while 23 unigenes, including BRI1s, BAK1s, TCH4, BIN2, and BZR1/2, were commonly upregulated in Sp, Su, and Au. Taken together, two-thirds of the 133 DEGs were activated in Su and Au (Supplementary Figure 6A). Thus, a number of BR-signaling factors (133 DEGs) had differential expression patterns among the different seasons in FD-TRs, suggesting that the signal transduction factors of BRs may play a role in SYQ FD-TRs, but their precise roles remain to be studied.

Cytokinins is one of the important phytohormones in controlling the initiation and expansion of TRs (Tanaka et al., 2008; Noh et al., 2010; Sojikul et al., 2015), but surprisingly, only 13 DEGs out of 92 CTK putative signal transduction factors, including homologs of the Arabidopsis histidine kinase 2/3/4 (cytokinin receptor, CRE1), histidine-containing phosphotransfer protein (AHP), two-component-response regulator B-ARR family (B-ARR), and two-component-response regulator A-ARR family (A-ARR), were identified in SYQ FD-TRs (Figure 9, Supplementary Table 8). About 9, including four A-ARRs, three B-ARRs, one AHP, and one CRE1, out of 13 DEGs were expressed at higher levels in Su and Au while AHP and B-ARR and two CRE1s were expressed at higher levels in Sp and Wi (Supplementary Figure 6H). These results suggest that the signal transduction factors of CTKs may not have an essential role in SYQ FD-TRs unlike their role during the fast growth stage of TR in other root crops (Wang Q. et al., 2006; Tanaka et al., 2008; Sun et al., 2015).

The signal transduction factors of ethylene are the second largest detected group. A total of 57 DEGs out of 134 unigenes were revealed, which include ethylene receptor (ETR), serine/threonine-protein kinase CTR1 (CTR1), mitogen-activated protein kinase 6 (MPK6), ethylene-insensitive protein (EIN), EIN3-binding F-box protein (EBF), and ethylene-responsive transcription factor (ERF) (Figure 9). A total of 40 out of the 57 DEGs were upregulated in Su. The numbers of DEGs upregulated in Au, Wi, and Sp were 20, 17, and 12, respectively (Supplementary Figure 6B). Ethylene promotes tuber formation by inhibiting GA biosynthesis and inhibiting elongation/cell expansion of the stem but stimulating tip swelling (Van De Poel et al., 2015). The EIN3 interacts with JA ZIM-domain-containing protein (JAZ) to positively regulate JA-dependent inhibition of primary root growth (Zhu et al., 2011), and several ERFs are regulated by EIN3, such as ERF1, ERF4, ERF9, ERF12, and EDF1 (Alonso and Stepanova, 2004) and are activated to inhibit root elongation but promote TR development (Firon et al., 2013; Sun et al., 2015; Li et al., 2019; Ding et al., 2020). These results suggest that ethylene signals may maintain SYQ FD-TRs for several years through the inhibition of vegetative reproduction and GA signals or by interacting with other signal transduction factors to improve the growth of TRs.

Auxin promotes TR formation at the early stage (Faivre-Rampant et al., 2004; Noh et al., 2010; Sojikul et al., 2015; Xu et al., 2016), but auxin content is subsequently decreased gradually during the secondary thickening growth of the TR (Wang Q. et al., 2006; Noh et al., 2010; Dong et al., 2019; Ding et al., 2020). Our analysis detected 55 DEGs of the 111 auxin signal unigenes, which include auxin influx carrier (AUX1), transport inhibitor response 1 (TIR1), auxin-responsive protein IAA (AUX/IAA), auxin response factor (ARF), auxin-responsive GH3 gene family (GH3), and small auxin-up RNA family protein (SAUR) (Figure 9, Supplementary Table 8). A total of 31 DEGs were expressed at higher levels in Sp or Wi, and 24 DEGs more abundant in Su, Sp, or Au (Supplementary Figure 6E). Among them, ARFs have been implicated in tuberization and the release of tuber dormancy in potato (Faivre-Rampant et al., 2004). These results suggest that the auxin signal transduction factors may play important roles in Sp to improve the growth of a new root and shoot, and to maintain the basal activities in SYQ FD-TRs in other seasons.

Gibberellins negatively regulate the development of TR and is an antagonist to auxin, ethylene, and ABA (Kloosterman et al., 2007). A total of 44 DEGs of 90 GA signal transduction factors were identified, which include gibberellin receptor GID1 (GID1), F-box protein GID2 (GID2), DELLA, phytochrome-interacting factor (PIF) (Figure 9, Supplementary Table 8), while 19 DEGs were expressed at higher levels in Sp, Wi, or both Sp and Wi. Noteworthily, 18 DEGs of the 35 DELLAs had different expression levels among at least two of the four seasons (Supplementary Figure 6C). DELLAs are GRAS family genes and negative regulators of GA signaling in multiple plant processes, including root development (Guo et al., 2014; Van De Velde et al., 2017), and have been shown to interact with more than 100 transcription factors (TFs) in *Arabidopsis* (Briones-Moreno et al., 2017) and multiple cascades of signaling hormones, including CTKs, ABA, BRs, auxin, ethylene, and JAs (Davière and Achard, 2016). These indicate that DELLA may be a central integrator of GA-dependent processes in a specific manner and may inhibit GA signaling in SYQ FD-TRs to block vegetative reproduction *via* interaction with ethylene, ABA, and their signal transduction factors (Kloosterman et al., 2007).

Secondary thickening growth of vascular cambium in TR is positively correlated with the concentrations of ABA and CTKs (Wang Q. et al., 2006; Tanaka et al., 2008; Sun et al., 2015; Ding et al., 2020). In SYQ FD-TRs, 33 DEGs, including abscisic acid receptor PYR/PYL family (PYP/PYL), serine/threonine-protein kinase SRK2 (SnRK2), and ABF, out of 68 ABA signaling unigenes were detected (Figure 9, Supplementary Table 8). About 14 DEGs, including PYP/PYLs, SnRK2s, and ABFs, were expressed at higher levels in Sp, Wi, or both Sp and Wi; 13 DEGs, including SnRK2s, ABFs, and PYP/PYL, were highly expressed in Su and Au; ABF12 and ABF18 in Wi, Su, and Au; and 3 SnRK2s and 1 PYP/PYL in Sp and Su (Supplementary Figure 6F). Among these unigenes, 10 ABFs were expressed at higher levels in FD-TRs of different seasons. *Arabidopsis* ABF4 and ABF2 proteins positively regulate potato tuber induction and maintain tuber dormancy (Muñiz García et al., 2014, 2018), and ABF4 has been shown to trigger a rise in ABA levels in stolons under tuber-inducing conditions (Muñiz García et al., 2014, 2018). Similarly, the expression level of *StABF1* is increased during tuber development, and StABF1's phosphorylation is stimulated by tuber-inducing conditions while GA inhibits StABF1 phosphorylation (Muñiz García et al., 2012). In addition, ABF4 is reported to increase the expression levels of *StGA2ox1* (coding a GA-inactivating enzyme) in stolons during tuberization (Muñiz García et al., 2014, 2018). These findings may indicate that activated ABFs in SYQ FD-TRs (Supplementary Figure 6F) may play an important role in the control of FD-TRs, and may interact with other signal factors such as GA, CTKs, ethylene, and auxin.

Jasmonic acid is involved not only in the production of secondary metabolites in plants, but also is a negative regulator of primary root growth (Ding et al., 2020), and a positive regulator for increasing the root diameter by inducing an expansion of cortex cells at the subapical region of the stolon (Koda et al., 2001; Cenzana et al., 2003; Ding et al., 2020). Out of 26 JA signal transduction factors, 19 DEGs, including JA-amino synthetase (JAR1), coronatine-insensitive protein 1 (COI1), JAZ, and TF MYC2 (MYC2), were detected (Figure 9, Supplementary Table 8). The JAR1, COI1-3, and two MYC2s expressed at higher levels in Sp and Wi; other 15 DEGs in Su and Au, and the most, including nine MYCs, five JAZs, and one COI, in Su (Supplementary Figure 6D). These results indicate that JA may also play some roles in the control of SYQ FD-TRs.

Salicylic acid (SA) is a key hormone in plant innate immunity, including both local and systemic tissues upon biotic attacks, hypersensitive responses, and cell death (Ding and Ding, 2020), and is also linked to the regulation of plant physiological and biochemical processes during the entire lifespan and abiotic stresses (Rivas-San Vicente and Plasencia, 2011). Of the predicted 26 SA signal unigenes, 15 DEGs, including regulatory protein NPR1 (NPR1), TF TGA (TGA), and pathogenesis-related protein 1 (PR-1), were identified (Figure 9, Supplementary Table 8). About 11 DEGs were expressed at higher levels in Su and Au, 10 DEGs, including PR-1, NPR1, and TGA, in Au, and 4 DEGs, including TGAs and NPR1, in Sp and Wi (Supplementary Figure 6G). Thus, SA may also be involved in response to biotic or abiotic stresses in SYQ FD-TRs.

### Validation of Some Genes by qPCR

To validate the expression profiles of DEGs obtained by RNA-Seq, 24 genes (Supplementary Figure 7) were selected for qPCR. First, we compared the expression levels of four housekeeping genes (Supplementary Table 1), and selected *UBC25F* as a control because it was constitutively expressed at similar levels in different TRs sampled at different times and different stages of development (data not shown). The expression levels of 21 out of the 24 genes were consistent with that of the RNA-Seq data (*R*^2^ = 0.643–0.930, and *p* < 0.05) (Supplementary Table 1). These indicate that our sampling method and RNA-Seq are suitable for studying the transcriptomic patterns and developmental processes of SYQ FD-TRs.

## Conclusion

The natural source of *T. hemsleyanum* has been decreased sharply due to its overexploitation for multi-applications, especially anti-cancer medicinal properties (Guo et al., 2019). However, the mechanisms of TR development and the biosynthesis pathways of valuable second metabolites in SYQ were scarcely known, which hindered an improvement in the cultivation technique. Hence, we investigated the changes of transcriptomic patterns in SYQ FD-TRs and detected putative highly enriched genes in the pathways of FP biosynthesis, starch and sucrose metabolism, and phytohormone signaling (Table 3, Figure 2). In this study, it was evident that the contents of total flavonoids (Supplementary Figure 5A) and some compounds of FPs (Figure 7) changed along with the expressions of some genes in their biosynthesis pathways (Figure 6, Supplementary Figure 7). The levels of these metabolites and expression of some genes in their biosynthesis pathways were higher in Au or Su, suggesting that Au is the best season for harvesting TR, and we also detected some genes in the FP biosynthesis that were induced significantly in Au, suggesting a role in FP biosynthetic pathways. Many genes in starch biosynthesis and degradation and in cellulose degradation pathway were more activated in Wi (Figures 8D–F) than the other seasons, indicating that the contents and kinds of starch and cellulose metabolites may play some roles in the control of FD-TRs in Wi. We detected for the first time that some phytohormone signal transduction factors belonging to BRs, auxin, JA, ethylene, ABA, GA, and CTKs in FD-TRs were activated in different seasons. With the exception of BRs, other hormones are considered as important regulators in the formation and development of TRs and tubers (Koda et al., 2001; Wang Q. et al., 2006; Noh et al., 2010; Sojikul et al., 2015; Ding et al., 2020). However, based on the numbers of DEGs in FD-TRs, BR signal transduction factors may play a pivotal role in the modulation of FD-TRs, and BRs may be applied in SYQ cultivation to improve its yield and quality. In addition, only some genes of the most gene families expressed differentially among the seasons in FD-TRs, suggesting that some genes in one family may have basal expressions, etc. have spatiotemporal differential expressions in FD-TRs or in other organs. However, we also observed some limitations to this study, for example, the lack of patterns in changes in sucrose, starch, and phytohormones, correlations between the expression level of genes in the biosynthesis or signaling pathways and the content patterns of these substances. Fewer sampling times may not produce conclusive findings, for instance, we only knew that Au is the best season for harvesting FD-TRs, but the optimal period may not be deduced from this result. These limitations will be addressed in future studies.

## Data Availability Statement

The original contributions presented in the study are publicly available. This data can be found at NCBI repository BioProject ID: PRJNA703059 (https://www.ncbi.nlm.nih.gov/bioproject/PRJNA703059).

## Author Contributions

WG and FT designed and supervised the experiments. QX, WG, SH, and JY collected the samples. QX, WG, and SH performed the majority of the experiments and prepared the tables and figures. AL-O organized the language and modified the manuscript. All authors contributed to the article and approved the submitted version.

## Conflict of Interest

The authors declare that the research was conducted in the absence of any commercial or financial relationships that could be construed as a potential conflict of interest.

## Abbreviations

2HIFD: 2-hydroxyisoflavanone dehydratase
4CL: 4-coumarate-CoA ligase
ABF: ABA responsive element binding factor
A/B-ARR family: A/B-ARR two-component response regulator
ABA: abscisic acid
AGPase: ADP-glucose pyrophosphorylase
AGS: α-glucosidase
AHP: histidine-containing phosphotransfer protein
AMY: α-amylase
ARF: auxin response factor
Au: autumn
AUX1: auxin influx carrier
AUX/IAA: auxin-responsive protein/indole-3-acetic acid
BAK: BRI-associated receptor kinase 1
BAM: β-amylases
BFF: beta-fructofuranosidase
BGLU: β-glucosidases
BIN2: protein brassinosteroid insensitive 2
BKI: BRI1 kinase inhibitor 1
BRs: brassinosteroids
BRI1: BR insensitive
BSK: BR-signaling kinase 1
BSU1: serine/threonine-protein phosphatase BSU1
C4H: cinnamate 4-hydroxylase
BZR1/2: BR resistant 1/2
CADG: cinnamyl-alcohol dehydrogenase
CAMT: caffeic acid 3-O-methyltransferase
CCOM: caffeoyl-CoA O-methyltransferase
CCR: cinnamoyl-CoA reductase
CEL3: endoglucanases
CHI: chalcone isomerase
CHS: chalcone synthase
COI1: coronatine-insensitive protein 1
CRE1: Arabidopsis histidine kinase 2/3/4 (cytokinin receptor)
CS3M: coumaroylshikimate 3′-monooxygenase
CSE: caffeoylshikimate esterase
CTKs: Cytokinins
CTR1: serine/threonine-protein kinase CTR1
CYCD3: cyclin D3
DEGs: differentially expressed genes
DPE: 4-α-glucanotransferases
EBF: EIN3-binding F-box protein
ECEL: cellulose 1,4-β-cellobiosidase
EIN: ethylene-insensitive protein
ERF: ethylene-responsive transcription factor
ETR: ethylene receptor
F3M: flavonoid 3′-monooxygenase
F6H: flavonoid 6-hydroxylase
F35H: N3H, flavonoid 3′ , 5′-hydroxylase
FD-TR: fully developed TR
FK: fructokinase
FLS: flavonol synthase
FPs: flavonoids and phenylpropanoids
GA: gibberellins
GBE: 1,4-α-glucan branching enzyme
GH3: auxin-responsive GH3 gene family
GID1: gibberellin receptor GID1
GID2: F-box protein GID2
GO: gene ontology
GPA: glucose-1-phosphate adenylyltransferase
HK: hexokinase
HPLC: high performance liquid chromatography
JA: jasmonic acid
JAR1: jasmonic acid-amino synthetase
JAZ: jasmonate ZIM-domain-containing protein
KEGG: Kyoto Encyclopedia of Genes and Genomes
MPK6: mitogen-activated protein kinase 6
MYC2: transcription factor MYC2
N3H: naringenin 3-dioxygenase
NPR1: regulatory protein NPR1
NR: nonredundant protein database
PAL: phenylalanine ammonia-lyase
PIF: phytochrome-interacting factor
PR-1: pathogenesis-related protein 1
PYP/PYL: abscisic acid receptor PYR/PYL family
RPKM: reads per kilobase per million
SAUR: small auxin-up RNA family protein
SHCT: shikimate O-hydroxycinnamoyltransferase
SnRK2: serine/threonine-protein kinase SRK2
Sp: spring
SPS: sucrose-phosphate synthase
SS: starch synthase
Su: summer
SUS: sucrose synthesis
SYQ: *Tetrastigma hemsleyanum* Diels et Gilg or Sanyeqing
TCH4: xyloglucan: xyloglucosyl transferase
TGA: transcription factor TGA
TIR1: transport inhibitor response 1
TR: tuberous root
UGP: UTP-glucose-1-phosphate uridylyltransferase
Wi: winter.
