# Supplementary material for: Seasonal Variation in Transcriptomic Profiling of Tetrastigma hemsleyanum Fully Developed Tuberous Roots Enriches Candidate Genes in Essential Metabolic Pathways and Phytohormone Signaling
Source: Front Plant Sci. 2021 Jul 9;12:659645. doi: 10.3389/fpls.2021.659645 (PMC8300961; doi:10.3389/fpls.2021.659645)
Supplement: Supplementary file 1 [file Data_Sheet_1.PDF]

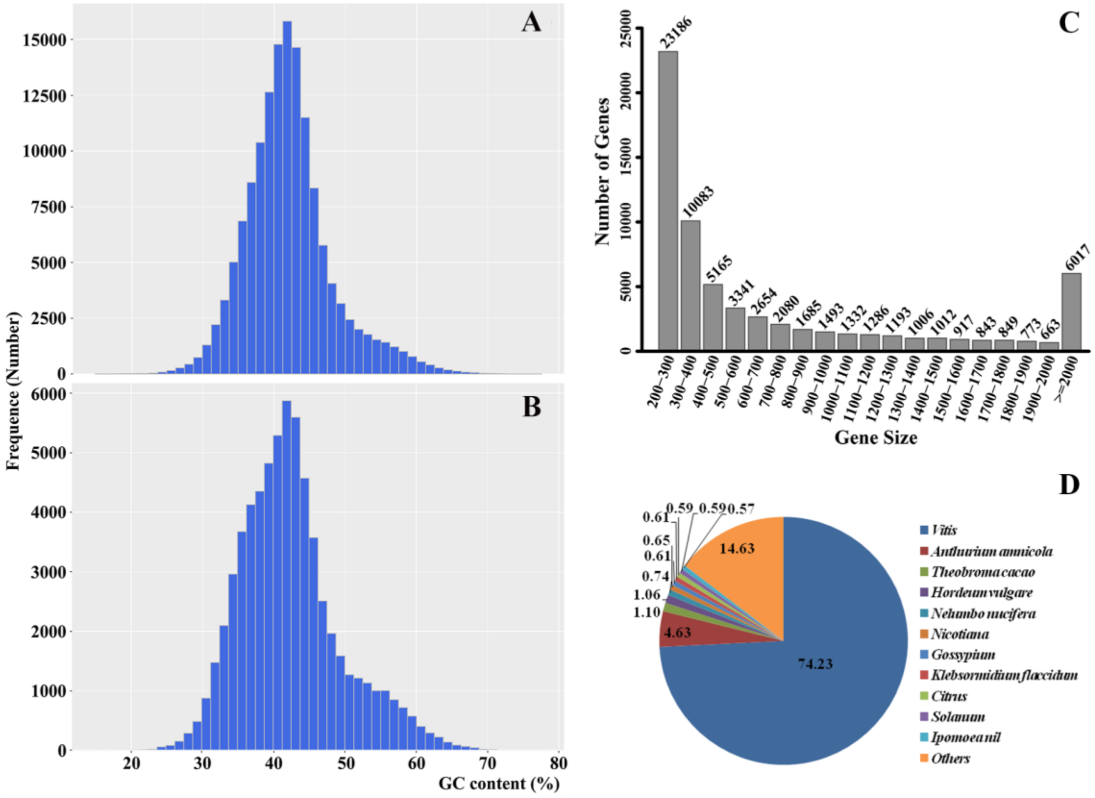

Supplementary Figure 1. Characteristics of transcripts and unigenes of *Tetrastigma hemsleyanum* fully developed tuberous roots. The distributions of GC contents in 144,096 transcripts (A) and 65578 unigenes (B), length distribution of 65578 unigenes (C), and distribution of NR annotated 35346 unigenes (BLASTx results) in different species (D).

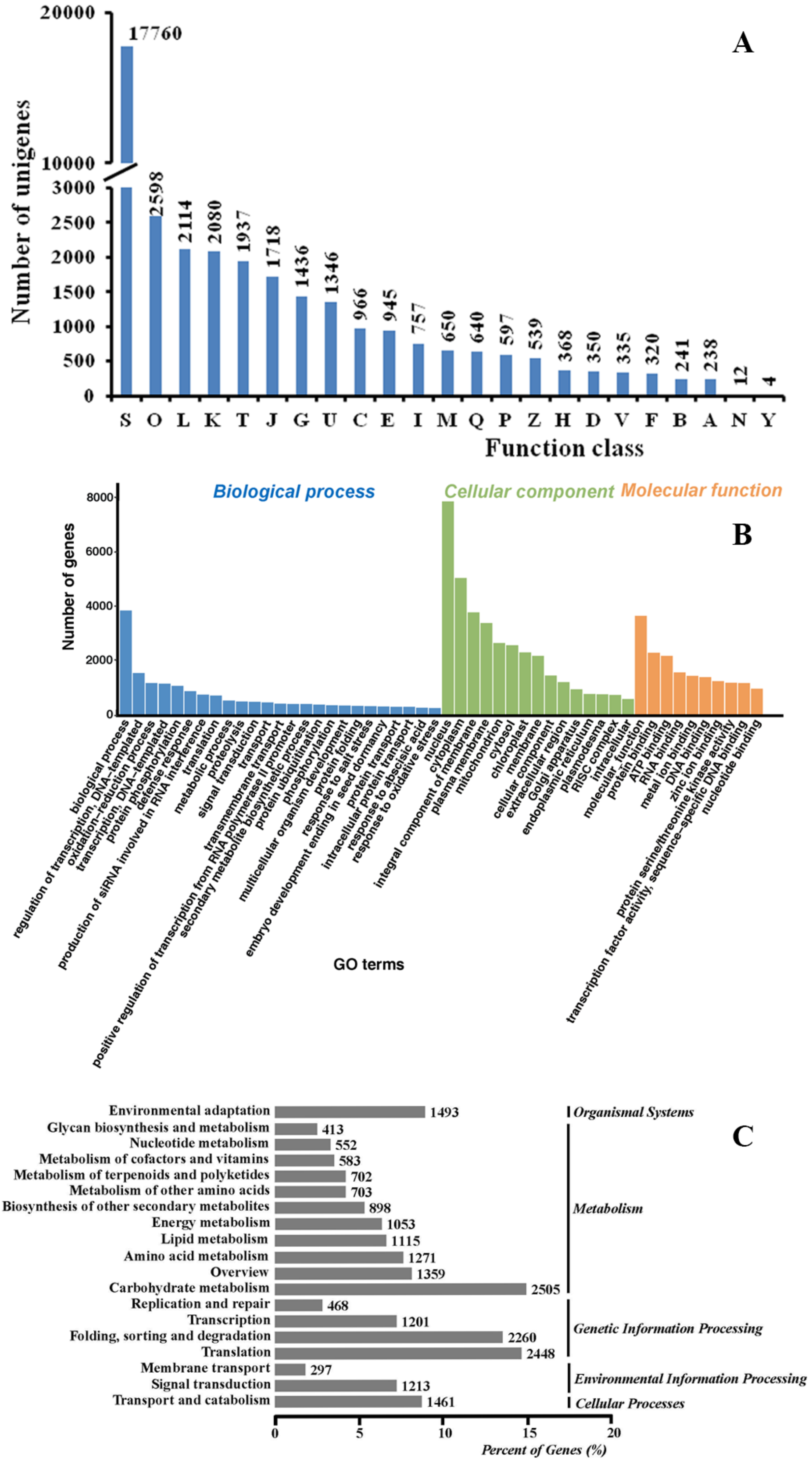
A
Number of unigenes
20000
10000
3000
2500
2000
1500
1000
500
0
17760
2598
2114
2080
1937
1718
1436
1346
966
945
757
650
640
597
539
368
350
335
320
241
238
12
4
S O L K T J G U C E I M Q P Z H D V F B A N Y
Function class
B
Biological process
Cellular component
Molecular function
Number of genes
8000
6000
4000
2000
0
biological process
regulation of transcription, DNA-templated
oxidation-reduction process
transcription, DNA-templated
protein phosphorylation
defense response
production of siRNA involved in RNA interference
translation
metabolic process
proteolysis
signal transduction
transport
transmembrane transport
positive regulation of transcription from RNA polymerase II promoter
secondary metabolite biosynthetic process
protein ubiquitination
phosphorylation
multicellular organism development
protein folding
response to salt stress
embryo development ending in seed dormancy
protein transport
intracellular protein transport
response to abscisic acid
response to oxidative stress
nucleus
cytoplasm
integral component of membrane
plasma membrane
mitochondrion
cytosol
chloroplast
membrane
cellular component
extracellular region
Golgi apparatus
endoplasmic reticulum
plasmodesma
RISC complex
intracellular
molecular function
protein binding
ATP binding
RNA binding
metal ion binding
DNA binding
zinc ion binding
protein serine/threonine kinase activity
transcription factor activity, sequence-specific DNA binding
nucleotide binding
GO terms
C
Environmental adaptation 1493
Glycan biosynthesis and metabolism 413
Nucleotide metabolism 552
Metabolism of cofactors and vitamins 583
Metabolism of terpenoids and polyketides 702
Metabolism of other amino acids 703
Biosynthesis of other secondary metabolites 898
Energy metabolism 1053
Lipid metabolism 1115
Amino acid metabolism 1271
Overview 1359
Carbohydrate metabolism 2505
Replication and repair 468
Transcription 1201
Folding, sorting and degradation 2260
Translation 2448
Membrane transport 297
Signal transduction 1213
Transport and catabolism 1461
Organismal Systems
Metabolism
Genetic Information Processing
Environmental Information Processing
Cellular Processes
0
5
10
15
20
Percent of Genes (%)

Supplementary Figure 2. Enrichment of unigenes of *Tetrastigma hemsleyanum* fully developed tuberous roots using eggNOG (A), GO (B), and KEGG (C) databases. S: Function unknown, O: Posttranslational modification, protein turnover, chaperones, L: Replication, recombination and repair, K: Transcription, T: Signal transduction mechanisms, J: Translation, ribosomal structure and biogenesis, G: Carbohydrate transport and metabolism, U: Intracellular trafficking, secretion, and vesicular transport, C: Energy production and conversion, E: Amino acid transport and metabolism, I: Lipid transport and metabolism, M: Cell wall/membrane/envelope biogenesis, Q: Secondary metabolites biosynthesis, transport and catabolism, P: Inorganic ion transport and metabolism, Z: Cytoskeleton, H: Coenzyme transport and metabolism, D: Cell cycle control, cell division, chromosome partitioning, V: Defense mechanisms, F: Nucleotide transport and metabolism, B: Chromatin structure and dynamics, A: RNA processing and modification, N: Cell motility, Y: Nuclear structure.

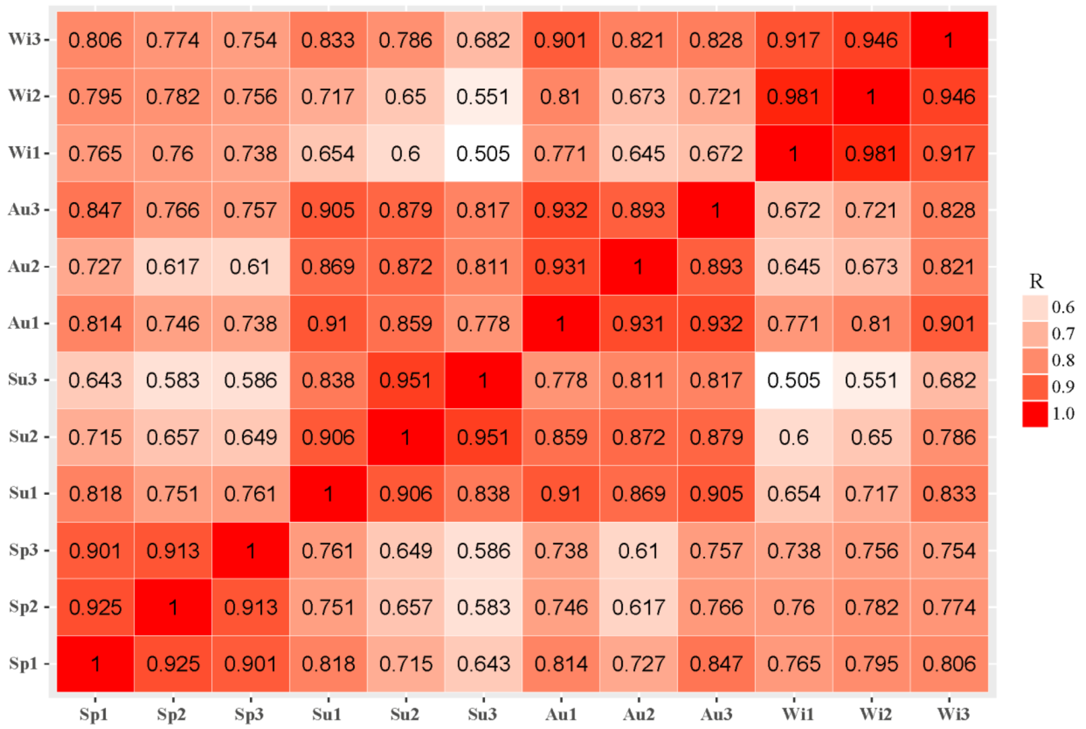

Supplementary Figure 3. Pearson correlation between 12 samples of *Tetrastigma hemsleyanum* fully developed tuberous roots using expression data of 65578 unigenes. Sp: spring; Su: summer; Au: autumn; Wi: winter.

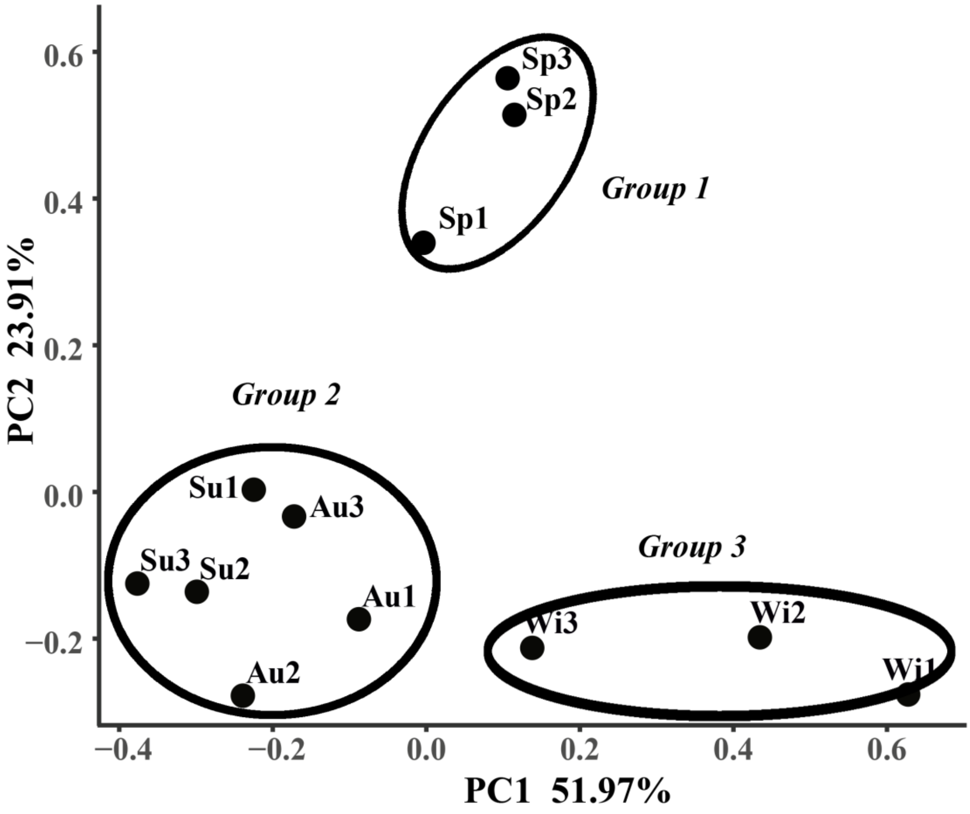

Supplementary Figure 4. Principal Component Analysis (PCA) of 12 samples *Tetrastigma hemsleyanum* fully developed tuberous roots using expression data of 65578 unigenes. Sp: spring; Su: summer; Au: sutumn; Wi: winter.

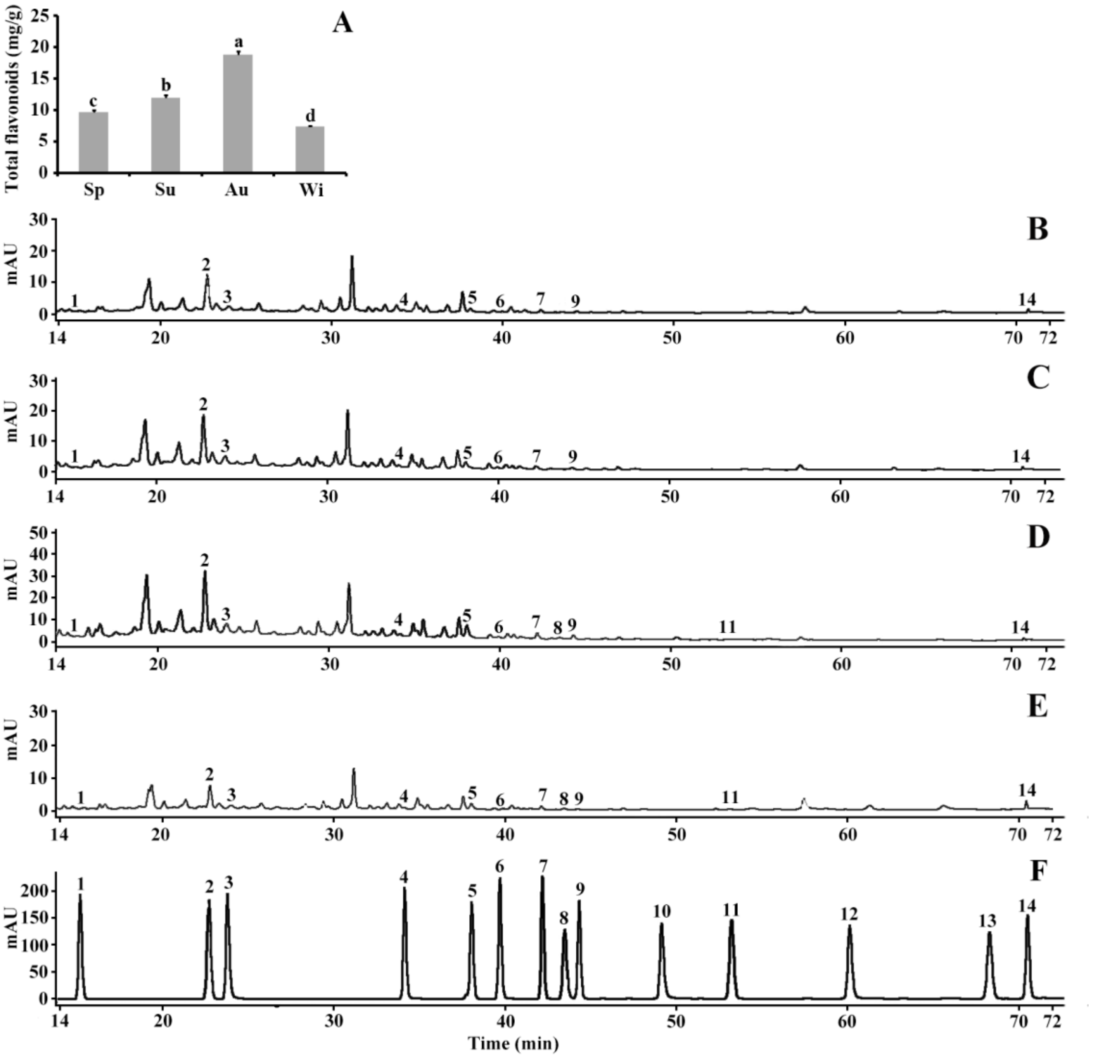

Supplementary Figure 5. Contents of total flavonoids (A) in *Tetrastigma hemsleyanum* fully developed tuberous roots and liquid chromatograms. (B) Sp, spring; (C) Su, summer; (D) Au, autumn; (E) Wi, winter; (F) 14 standard-compound mixtion. 1, protocatechuic acid; 2, catechin; 3, chlorogenic acid; 4, orientin; 5, polydatin, 6, isoquercitrin; 7, nictoflorin; 8, piceatannol; 9, astragaline; 10, myricetin; 11, resveratrol; 12, quercetin; 13, naringenin; and 14, kaempferol. Bars represent means + SE, (n = 5).

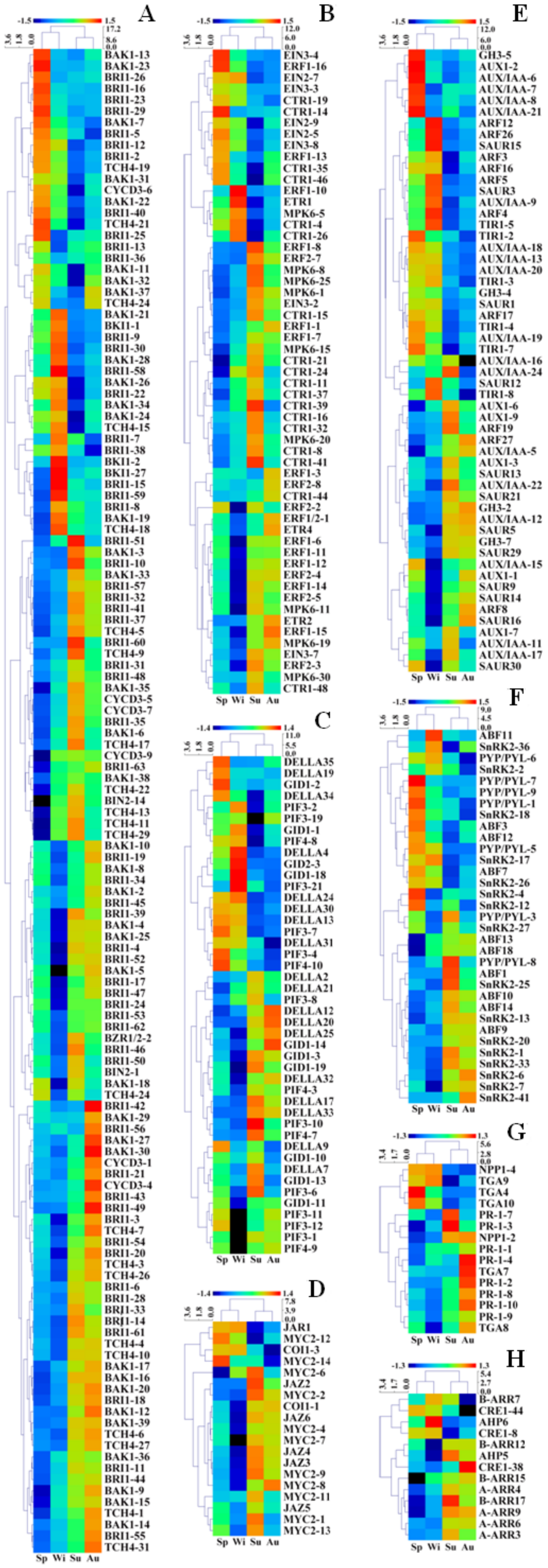

Supplementary Figure 6. Heatmap represents expression dynamics of genes in 8 phytohormone signal transduction pathways in *Tetrastigma hemsleyanum* fully developed tuberous roots. A, Brassinosteroid, B, Ethylene, C, Gibberellin, D, Jasmonic acid, E, Auxin, F, Abscisic acid, G, Salicylic acid, and H, Cytokinine. AUX1, auxin influx carrier; TIR1, transport inhibitor response 1; AUX/IAA, auxin-responsive protein IAA; ARF, auxin response factor; GH3, auxin responsive GH3 gene family; SAUR, SAUR (small auxin-up RNA) family protein; CRF, arabidopsis histidine kinase 2/3/4 (cytokinin receptor); AHP, histidine-containing phosphotransfer peotein; ARR-A, two-component response regulator ARR-A family; ARR-B, two-component response regulator ARR-B family; GID1, gibberellin receptor GID1; GID2, F-box protein GID2; PIF, phytochrome-interacting factor; PYL, abscisic acid receptor PYR/PYL family; SnRK2, serine/threonine-protein kinase SRK2; ABF, ABA responsive element binding factor; ETR, ethylene receptor; CTR1, serine/threonine-protein kinase CTR1; MPK6, mitogen-activated protein kinase 6; EIN2, ethylene-insensitive protein 2; EIN3, ethylene-insensitive protein 3; EBF1/2,EIN3-binding F-box protein; ERF; ethylene-responsive transcription factor; BAK1, brassinosteroid insensitive 1-associated receptor kinase 1; BRI1, protein brassinosteroid insensitive 1; BKI1, BRI1 kinase inhibitor 1; BSK, BR-signaling kinase; BSU1, serine/threonine-protein phosphatase BSU1; BIN2, protein brassinosteroid insensitive 2; BZR1/2, brassinosteroid resistant 1/2; TCH4, xyloglucan:xyloglucosyl transferase TCH4; CYCD3, cyclin D3; JAR1, jasmonic acid-amino synthetase; COI-1, coronatine-insensitive protein 1; JAZ, jasmonate ZIM domain-containing protein; MYC2, transcription factor MYC2; NPR1, regulatory protein NPR1; TGA, transcription factor TGA; PR-1, pathogenesis-related protein 1. Sp, spring; Su, summer; Au, autumn; Wi, winter.

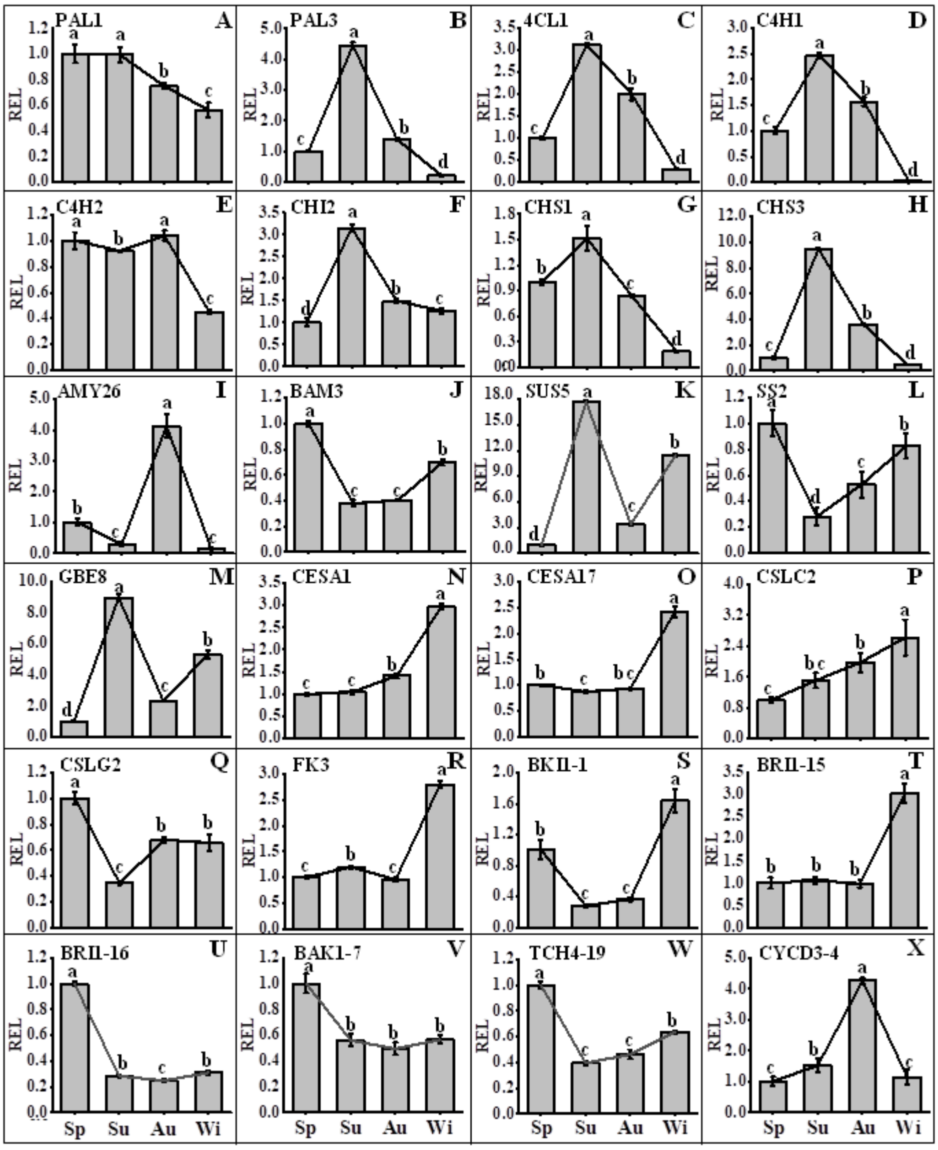

Supplementary Figure 7. Validation of the predicted RNA-seq genes using qPCR in *Tetrastigma hemsleyanum* fully developed tuberous roots. Bars represent means + SE, (n = 3). Bars bearing same letter are not significantly different. Genes A-H involved in flavonoid and phenylpropanoid biosynthesis; genes I-R involed in polysaccharide metabolism; and genes S-X involved in plant hormone signal transduction. The information of those genes is refer to Table S1. A significance threshold of $P < 0.05$ was considered statistically significant. PAL, phenylalanine ammonia-lyase; 4CL, 4-coumarate--CoA ligase; C4H, trans-cinnamate 4-monooxygenase; CHI, chalcone isomerase; CHS, chalcone synthase; AMY, α-amylase; BAM, beta-amylase; SUS, sucrose synthase; SS, starch synthase; GBE, 1,4-α-glucan branching enzyme; CESA, 1,4-beta-D-xylan synthase; CSLC, xyloglucan glycosyltransferase; CSLG, Cellulose synthase-like protein; FK, fructokinase; BKI, BRI1 kinase inhibitor; BRI, brassinosteroid insensitive; BAK, brassinosteroid insensitive 1-associated receptor kinase; TCH, yloglucan:xyloglucosyl transferase; CYCD, Cyclin-D; REL, Relative expression level; Sp, spring; Su, summer; Au, autumn; Wi, winter.
